# All optical control of magnetization in quantum confined ultrathin magnetic metals

**DOI:** 10.1038/s41598-021-95319-6

**Published:** 2021-08-05

**Authors:** Saeedeh Mokarian Zanjani, Muhammad Tahir Naseem, Özgür Esat Müstecaplıoğlu, Mehmet Cengiz Onbaşlı

**Affiliations:** 1 Graduate School of Materials Science and Engineering, Koç University, Sarıyer, 34450 Istanbul, Turkey; 2 Department of Physics, Koç University, Sarıyer, 34450 Istanbul, Turkey; 3 Department of Electrical and Electronics Engineering, Koç University, Sarıyer, 34450 Istanbul, Turkey

**Keywords:** Condensed-matter physics, Magnetic properties and materials, Spintronics

## Abstract

All-optical control dynamics of magnetization in sub-10 nm metallic thin films are investigated, as these films with quantum confinement undergo unique interactions with femtosecond laser pulses. Our theoretical analysis based on the free electron model shows that the density of states at Fermi level (DOS_F_) and electron–phonon coupling coefficients (G_ep_) in ultrathin metals have very high sensitivity to film thickness within a few angstroms. We show that completely different magnetization dynamics characteristics emerge if DOS_F_ and G_ep_ depend on thickness compared with bulk metals. Our model suggests highly efficient energy transfer from femtosecond laser photons to spin waves due to minimal energy absorption by phonons. This sensitivity to the thickness and efficient energy transfer offers an opportunity to obtain ultrafast on-chip magnetization dynamics.

## Introduction

Quantum confined magnetic nanomaterials such as magnetic ultrathin metals and alloys, and diluted magnetic semiconductors (DMS), provide rich emerging new physics^1–3^. There is also significant research on the quantum confinement effect in the atomic thin semiconductors for novel spin-based photonic quantum technologies and applications^4^. Metallic magnetic thin films have been investigated in applications such as femtosecond (fs) laser pulse switching of magnetization^5–8^. Elemental magnetic metals with low spin–orbit coupling are ideal for this purpose. The mechanism of all-optical switching (AOS) of magnetization includes the electron bath thermalization after illumination by a fs laser pulse, followed by spin and phonon baths coupling with electrons via the electron–phonon coupling. Magnetic metallic ultrathin films (thicknesses less than 10 nm) behave differently to the fs laser pulse because of the change in the density of state at the Fermi level due to the quantum confinement^1,9,10^. The free-electron theory of metals provides the opportunity to understand the quantum effects associated with the film thickness. Because of its simplicity, the thin-film quantum well is appropriate and provides an introductory justification to the quantum size effects^11^. This effect directly changes the electron heat capacity constant (known as Sommerfeld coefficient, γ) and coupling between electron and phonon. This concept is defined as all-optical quantum manipulation of magnetization.

**Figure 1:**
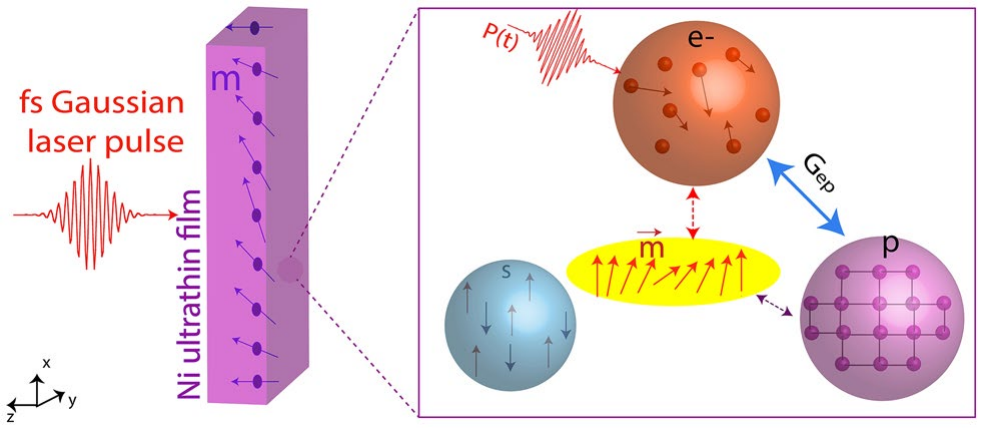
(Left) The schematic of ultrashort (femtosecond) laser pulse interaction with the metallic magnetic ultrathin film. (Right) Coupled interaction between electron, phonon, and magnetization is shown. We investigate the energy transfer by employing extended microscopic three temperature model.

In this study, we theoretically investigate the magnetization dynamics for sub-10 nm isolated, free-standing metallic thin films after exposure to a femtosecond laser pulse, the schematic description of which is shown in Fig. 1. The laser pulse power is directly transferred to the electron bath, and the electron temperature (T_e_) increases quickly in the sub-picosecond timescale. Electron thermalization results in a sharp decrease in the magnetization of the thin film. Due to the electron–phonon coupling, as shown on Fig. 1, T_e_ balances its energy with a phonon bath, and reaches thermal equilibrium. The magnetization of the film is recovered in the following picoseconds. This energy transfer has been studied with microscopic three temperature model (M3TM)^12,13^. Due to the lack of more rigorous or quantum approaches, we stick to the M3TM as a clearly non-ideal but rather illustrative one for describing the ultrafast laser-magnetism interaction in quantum-confined nanometals. To investigate the quantum confinement effects on the magnetization dynamics, first, we calculate the electron density of state at Fermi level (DOS_F_), electron–phonon coupling coefficient (G_ep_), Sommerfeld coefficient (γ), and magnetization dynamics in quantum-confined magnetic metals using M3TM^14,15^. Then, we analyze the variability of magnetization dynamics as a function of film thickness. Previous studies investigated the laser light interaction with magnetic material^16–19^, however, quantum effects associated with the film thickness on the magnetization dynamics have not been examined.

In this paper, we used M3TM as a tool to show quantum size oscillations manifested in observables like magnetization. The idea of the quantum size effect on the electron–phonon coupling will lead to additional theoretical and experimental in the field of laser-induced spintronic phenomena. The coefficients used to solve M3TM differential equations are derived from microscopic Hamiltonians, such as Frochlich electron–phonon interaction.

Other studies report various micromagnetic models to describe laser-induced magnetization dynamics, which exhibit a similar structure of rate of equations^20–27^. Manchon et al.^22^ propose a microscopic theory of the laser-induced magnetization dynamics under the three-temperature framework and derive the equations that govern the demagnetization at arbitrary temperatures. A self-consistent random phase approximation is developed and a set of dynamic equations for the time-dependent temperatures of electrons, spins, and lattice are explicitly expressed in terms of the microscopic parameters. The resulting equations are similar to the phenomenological three-temperature model. Similarly, a self-consistent spin-phonon dynamical model based on the LLB equation and the quantum version of LLB (qLLB)^23–25^, as useful methods to model interesting phenomena where the magnetic and temperature dynamics are relevant are proposed. These models consider the dependence of the magnetization dynamics on bath temperatures using a simple spin-phonon Hamiltonian, which is also valid for simple spin-electron Hamiltonian. Another approach based on a many-body pd-band Hamiltonian^26^ predicts that the degree of demagnetization correlates with the average number of electrons excited by the laser or the average number of absorbed photons. This study suggests that the laser-induced ultrafast demagnetization effect could be used in ferromagnetic small clusters, nanoparticles and granular systems to reveal the size and structural dependence. Moreover, microscopic theory of ultrafast out-of-equilibrium magnon-phonon dynamics in insulators^27^ is explained by the energy transfer between the phonon, and spin baths and the induced change of phonon populations is calculated based on the Fermi’s Golden rule calculating the scattering terms and coupled energy rate equations.

These studies provide resembling rate equations with common characteristic dependences on microscopic interaction coefficients. Therefore, similar quantum size oscillations could be expected by using different models.

The magnetization behavior is captured by the Landau-Lifshitz-Bloch equation (LLB)^24^, which describes the time and temperature dependent change of the magnetization after interaction with a fs laser pulse. This model, though, does not study the energy balance between the electron and phonon baths. M3TM does not include the coupling of spins with electrons and phonons. The magnetization dynamics is influenced by the energy balance parameters such as G_ep_, γ, and spin-flip ratio (R) determining the timescales of magnetization change. A more comprehensive model is needed to include both magnetization dynamics and electron (phonon) bath equilibrium.

In many magnetic materials, weak spin–orbit interactions significantly reduce spin-electron and spin-phonon scattering. Magnetic metallic systems with large spin–orbit coupling, such as transition metal interfaces, 2D electron gas, or emergent phenomena such as SrTiO_3_/LaTiO_3_ interfaces which yield emergent superconductivity and large spin–orbit coupling^28^, and also transition metal dichalcogenides^29–31^ are excluded from the scope of this study. Our model here is advantageous due to eliminating some scattering events such as spin-phonon and spin electron scattering^32^.

## Results

### Microscopic three temperature model (M3TM) and magnetization dynamics

For our model shown in Fig. 1, we solve the M3TM including magnetization dynamics (extended M3TM) in the Eqs. (1)–(3) based on Koopmans’s model^5^.

1 $${\mathrm{C}}_{\mathrm{e}}\frac{{\mathrm{dT}}_{\mathrm{e}}}{\mathrm{dt}}=-{\mathrm{G}}_{\mathrm{ep}}\left({\mathrm{T}}_{\mathrm{e}}-{\mathrm{T}}_{\mathrm{p}}\right)+\mathrm{P}\left(\mathrm{t}\right)$$

2 $${\mathrm{C}}_{\mathrm{p}}\frac{{\mathrm{dT}}_{\mathrm{p}}}{\mathrm{dt}}=-{\mathrm{G}}_{\mathrm{ep}}\left({\mathrm{T}}_{\mathrm{p}}-{\mathrm{T}}_{\mathrm{e}}\right)$$

3 $$\frac{\mathrm{dm}}{\mathrm{dt}}=\mathrm{Rm}\frac{{\mathrm{T}}_{\mathrm{p}}}{{\mathrm{T}}_{\mathrm{C}}}\left(1-\mathrm{mcoth}\left(\mathrm{m}\frac{{\mathrm{T}}_{\mathrm{C}}}{{\mathrm{T}}_{\mathrm{e}}}\right)\right)$$

where T_e_ (T_p_) is electron (phonon) temperature, and m is the magnetization. The details of the rest of the parameters appearing in Eqs. (1)–(3) is given in Table 1. These differential equations describe the energy transfer from femtosecond laser pulse P(t) to electron, phonon, and magnetization (m =|M_z_|/M_s_). The spin-flip ratio (R) in Eq. (3) is a parameter that determines the kinetics of the transient magnetization change. According to Ref.^5^ the spin-flip ratio depends on G_ep_ which further depends on the DOS_F_ and thickness of the ultrathin film L_z_.

**Table 1:** Parameters used in extended M3TM.

Parameter	Explanation	Value
C_p_	Heat capacity of phonon	2.33** × **10^6^ (J m^−3^ K^−1^)^5^
C_e_	Heat capacity of electron (dependent of electron temperature (T_e_))	C_e_ = γ T_e_ (J m^−3^ K^−1^)^5^
γ	γ _0_·DOS_F_	Calculated in the methods section and shown in Fig. 2 (J m^−3^ K^−2^)^1^
γ _0_	Sommerfeld coefficient (C_p_/5T_C_)	743.22 (J m^−3^ K^−2^)^5^
G_ep_	G_0_·DOS_F_	Thickness-dependent e-p coupling coefficient (see the methods section and Fig. 2)^1^
G_0_	(πK_B_/ħ)·λ(ω^2^)	Calculated in the methods section and shown in Fig. 2 (W m^−3^ K^−1^)^1^
R	R = spin-flip ratio = R_0_ × DOS_F_	17.2** × **10^12^ (s^−1^)^5^^,32^
T_C_	Curie temperature of Ni	627 (K)^5^

We consider the incoming laser pulse power as a Gaussian single pulse per unit volume as

4 $$\mathrm{P}\left(\mathrm{t}\right)=\frac{{\mathrm{P}}_{0}}{\sqrt{2\uppi }}\mathrm{exp}\left(\frac{-1}{2}{\left(\frac{\mathrm{t}}{{\mathrm{t}}_{0}}\right)}^{2}\right)$$

where P_0_ = $\frac{{I}_{0}}{d \cdot {t}_{0}}$, I_0_ and t_0_ are the laser pulse fluence in J m^−2^ and pulse width (fs), respectively. The injected laser fluence is normalized to a fixed thickness d, to capture the pulse energy per unit volume.

We have considered simplifications in the differential equations which describe the electron and phonon temperature profile. Immediately after the illumination by the sub-picosecond laser pulse, two competing processes occur at the non-equilibrium state of the excited electrons. The non-thermalized electrons move with a velocity close to the Fermi velocity and continue to thermalize into a Fermi–Dirac distribution through collision. It takes a finite time for the excited electrons to travel and complete the thermalization^33^. But in our study, the electron thermalization time is considerably shorter than the laser pulse duration. Moreover, for sub-picosecond pulses, the laser energy is primarily absorbed by the free electrons on the film's surface. Most of the electron thermal energy is then transferred to the lattice; meanwhile, another part of the energy diffuses to the electrons in the deeper sub-surfaces. Because the pulse duration is too short, the laser is turned off before thermal equilibrium between the electrons and lattice is reached^34^. In fact, on the picosecond time scale, longitudinal temperature gradients and transverse heat propagation are neglected due to the small sample thickness^16^.

To the best of our knowledge, there is no former experimental or theoretical study on all-optical control of magnetization dynamics of quantum confined ultrathin metals, which considers the thickness dependence of G_ep_ and γ in the extended M3TM. Due to the low thickness of the thin films and quantum confinement effects, the electron–phonon coupling (G_ep_) changes dramatically with the film thickness in the nanometer (nm) regime. However, there is an experimental report on the effect of thickness on the laser induced demagnetization time of the Co/Pd multilayers with a few nm thickness of each layer, by measuring the Kerr signal of different laser excitation and a various number of layers^35^.

Due to the quantum confinement in the electron density of states at the Fermi level, both the electron–phonon coupling and Sommerfeld coefficient cannot be considered constant, which we have included these confinement effects in our analysis accordingly. However, our analysis is based on several assumptions (see “Methods”) which are justified in the considered parameters. One of the important assumptions is neglecting the size effects on phonon bath by using the typical bulk value of phonon specific heat. In finite-size systems, such as thin metallic films, confinement effects could influence the thermal properties of the lattice phonons. Size effects are most significant for thermal transport^36^, on contrary, thermodynamic properties, such as specific heat, do not change significantly with the size provided the temperatures of interest are much lower than the Debye temperature. The primary effect of the finite size is to have fewer states to count in the calculation of specific heat. In strict mathematical terms, the continuum approximation to the states in the k-space cannot be made in the finite size systems, and the sum over the states cannot be replaced by an integral. However, at low temperatures, the ratio of the exact specific heat determined by the summation to the integral is close to one. For example, it is about ∼ 0.9856 for Aluminum thin film, using the Debye model phonon dispersion relation^37^. Accordingly, neglecting the size effects on phonon bath by using the typical bulk value of phonon specific heat is a reasonable approximation in our calculations.

### Effect of film thickness (L_z_) on chemical potential (µ), Fermi level electronic density of states (g_F_), electron–phonon coupling (G_ep_), and Sommerfeld coefficient (γ)

In our numerical calculations, we solve Eqs. (1)–(3) which is based on Koopmans’s model^5^, for experimentally available Ni thin-film parameters (see Table 1). In this study, we use the laser fluence of 28 mJ m^−2^ and the pulse duration of 50 fs.

**Figure 2:**
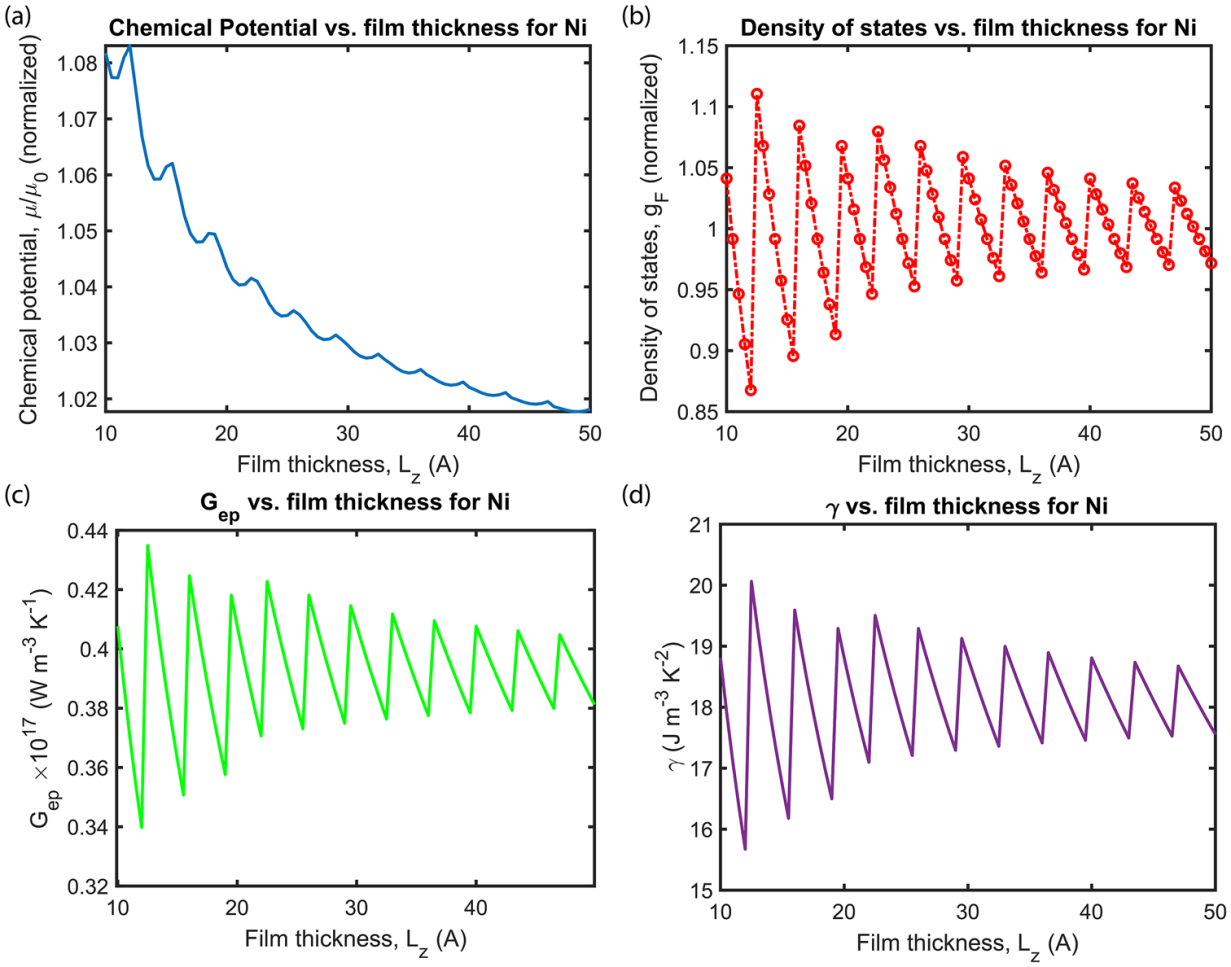
Thickness dependent quantum-confinement effect. (**a**) Chemical potential µ/µ_o_ (finite temperature Fermi energy), (**b**) density of states at the Fermi energy g_F_, (**c**) electron–phonon coupling coefficient G_ep_, and (**d**) Sommerfeld coefficient γ as a function of ultrathin film thickness L_z_. Both µ/µ_o_ and g_F_ are dimensionless, normalized with their corresponding bulk values. The parameters are considered of Ni thin film and details are given in Table 1.

In Fig. 2a,b, L_z_-dependence of chemical potential µ and DOS_F_ at Fermi energy are shown, respectively. Both µ and g_F_ are dimensionless, normalized with their corresponding bulk values. We consider parameters for Ni, which has λ⟨ω^2^⟩ = 49.5 meV^2^^1^, and it is converted to J^2^ in the numerical calculations. The depth of the metallic confinement potential is assumed to be V_z_ = 10 eV. The V_z_ value is theoretically infinite, however, in the free electron model (FEM) simulations, its value is considered as a finite number comparable to its bulk Fermi value^10^.

The FEM perfectly reflects the oscillations in the DOS_F_, G_ep,_ and C_e_ as the result of quantum confinement for the metals whose valance electrons lie in p-band such as Al. Since Ni is a transition metal with an almost full d-band structure^1^, the possible complexity in the configuration of electrons in the Fermi level would require beyond free electron methods to determine the DOS_F_, such as density functional theory (DFT). However, FEM still captures the essential physics behind the quantum size effects even for transition metals and more complex structures^11^. Ab initio methods confirm the conclusions of the free electron model^38,39^.

The oscillations of G_ep_ (Fig. 2c) and γ (Fig. 2d) are due to the Fermi level oscillations translated to DOS_F_, arising from the quantum confinement (cf. Eq. (8) in “Methods”). This essential physics (discreteness of the k_z_) remains the same irrespective of the simplicity or complexity of the Fermi surface. Failure of the continuum approximation in the confined direction yields discrete plateaus for the electronic states. Accordingly, the formation of such quantum well states (QWS) in the confined direction captures the basic physics of the quantum size effects, manifested as size-dependent oscillations in physical observables^40–43^. We show the Fermi level oscillations in Fig. 2c, which translates to DOS_F_, hence to G_ep_ and C_e_. For Ni, although the d-band structure leads to a more complicated Fermi level than Al, which decreases the magnitude of G_ep_ compared to the values used in the experimental studies^1^. Even if the decrease in these terms is not entirely in agreement quantitatively with the reported values, our model still reflects the effect qualitatively. FEM predicts quantum size effect oscillations in the magnetization but should be regarded as a qualitative description for the magnetic metals with more complex Fermi configurations such as Ni. The effect can be studied more rigorously and quantitatively using ab initio calculations of the band structure, Fermi level, and DOS_F_. Furthermore, it can be optimized by considering more complex materials using beyond FEM analysis. In this work, we will be presenting the essential physics and predicting quantum size effect in magnetization in the same spirit of quantum size effects in electronic conduction.

### Microscopic three temperature model coupled with magnetization dynamics

**Figure 3:**
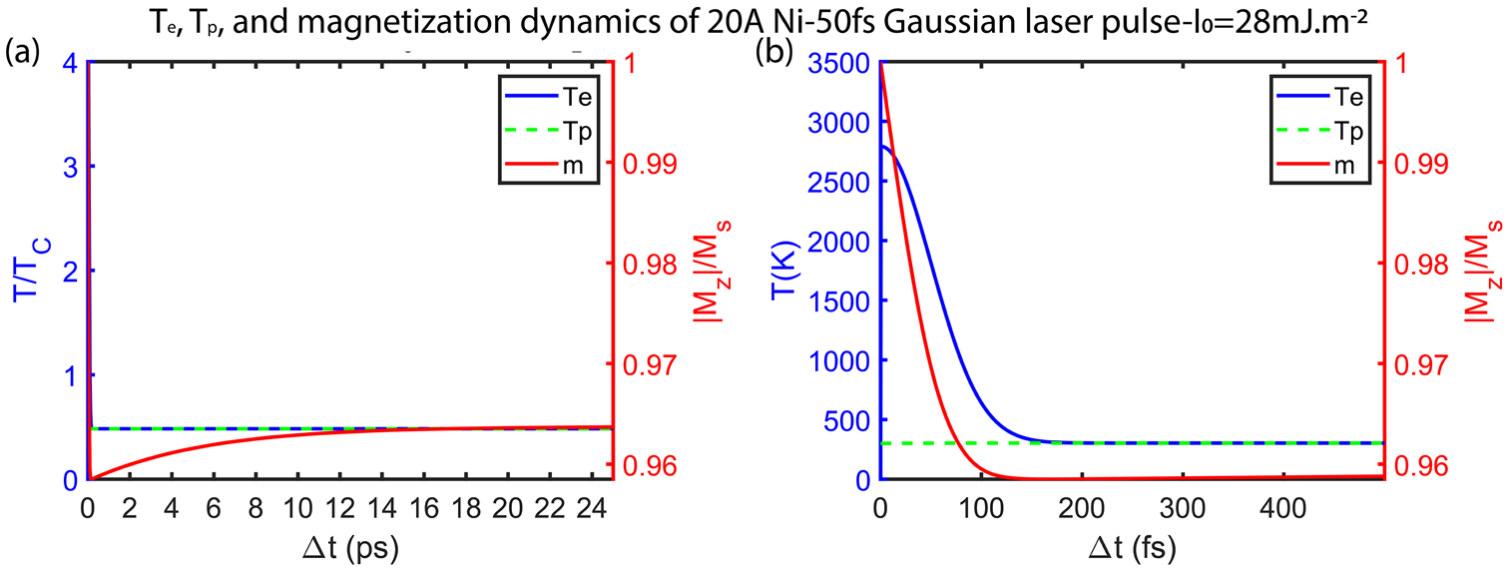
(**a**) Transient T_e_, T_p_, and normalized magnetization (m =|M_z_|/M_s_) for 20 Å thick Ni film illuminated with I_0_ = 28 mJ m^−2^ Gaussian single laser pulse of 50 fs width. (**b**) Zoomed-in version of (**a**) 500 fs after illumination. Note that the Y-axis units are different in (**a**) and (**b**).

Here, we discuss the quantum confinement effect on the magnetization dynamics for Ni thin film. In Fig. 3, the magnetization dynamics and transient electron and phonon temperatures calculated from the extended M3TM are shown for 20 Å Ni thin film. According to Fig. 3, illuminated with a Gaussian single laser pulse of I_0_ = 28 mJ m^−2^, the magnetization of the Ni thin film decreases in sub-100 fs due to the thermalization of the electron bath and E-Y scattering. Due to the low heat capacity of the electrons, T_e_ reaches 1.5T_c_ of Ni (940.5 K). However, due to electron–phonon coupling, T_e_ cools down to an equilibrium temperature with phonon (lattice) in 200 fs, and the magnetization recovers to close to its initial value (> 96%), in around 1 ps. Note that T_p_ in Fig. 3b is not constant (since the magnetization recovery is not completed in 500 fs), and its increasing rate is very low compared to T_e_ and it is not completely visible in Fig. 3b (T_p_(100 fs) = 302.3 K, T_p_(500 fs) = 302.7 K).

Note that Fig. 3b shows the temporal change of the magnetization, electron, and phonon temperatures, before reaching the equilibrium (which occurs 15 ps after laser pulse incidence). The sudden rise of the T_e_, due to the lower electron heat capacity, in the non-equilibrium state is in the range of a few fs.

We also calculated the effect of the pulse width on the magnetization dynamics and electron and phonon temperature. The results are shown in the supplementary information Figs. S9–S11. Increasing the pulse width decreases the laser power injected to the thin film which leads to an increase in the demagnetization time as well as electron equilibration time. Choosing very long pulse durations, suppresses the injected laser power and increases the laser fluence needed for recovery of the magnetization after quenching, which is not favorable for the scope of our manuscript. In addition, we have considered the condition where the incoming laser fluence is in the range of the experimental values, with only 20% absorption in the quantum confined thin film. We compared the results of M3TM for the nm-thick non-quantum confined and angstrom-thick quantum confined Ni film, the results of which are shown in supplementary Fig. S12. For more extensive investigation, we also compared the effect of laser pulse width (both in fs and ps regimes) on the temporal behavior of the magnetization, electron, and phonon temperatures in nm-thick and angstrom-thick Ni film, in supplementary Figs. S13 and S14.

### Dependence of the magnetization dynamics on film thickness (L_z_)

**Figure 4:**
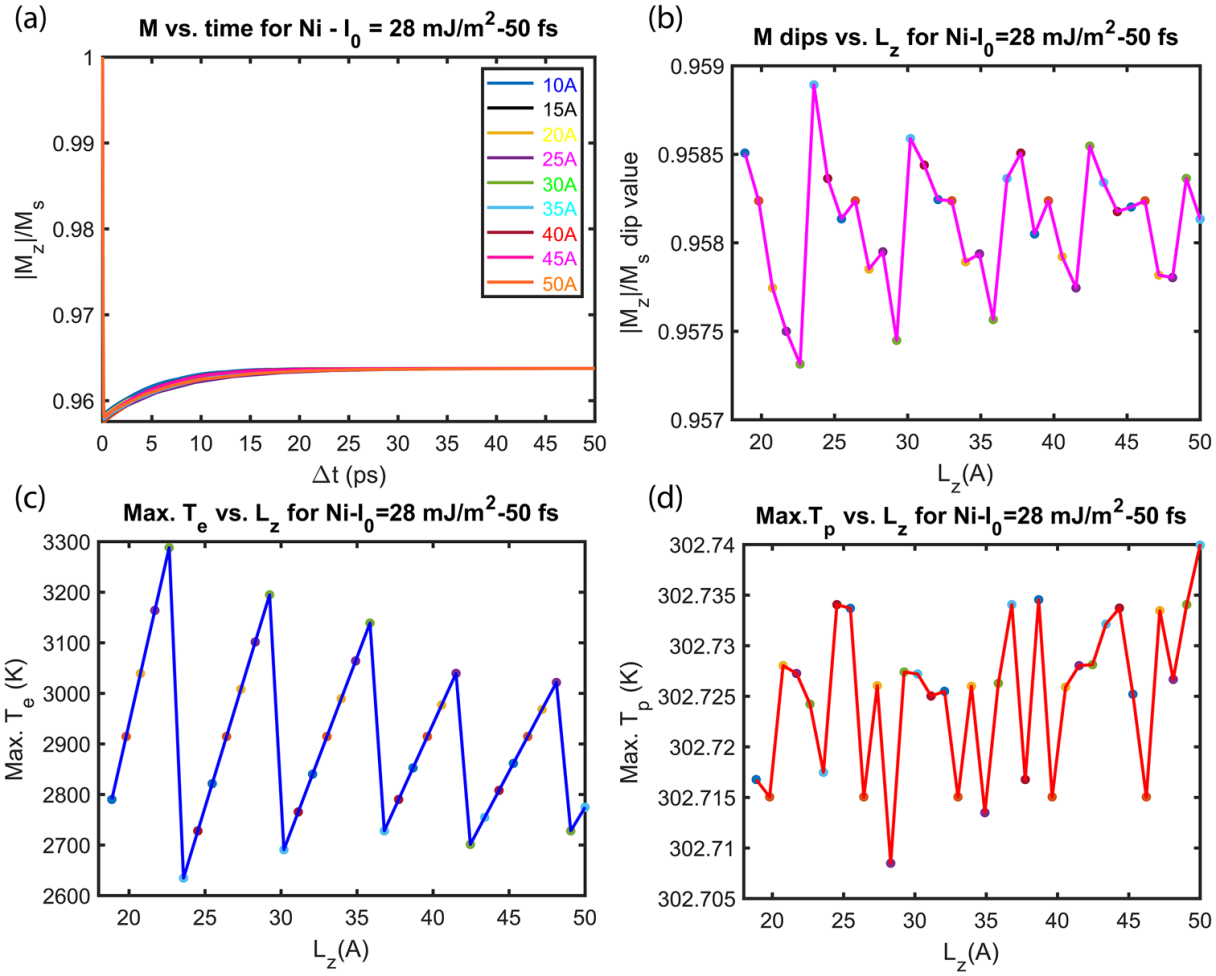
Effect of Ni film thickness L_z_ on (**a**) magnetization dynamics, (**b**) demagnetization dip, (**c**) maximum of electron temperature T_e_, and (**d**) maximum of phonon temperature T_p_ for L_z_ = 10 Å, 15 Å, 20 Å, 25 Å, 30 Å, 35 Å, 40 Å, 45 Å, and 50 Å. The Ni thin film is illuminated with I_0_ = 28 mJ m^−2^ Gaussian single laser pulse. The rest of the parameters are given in Table 1. The zoomed-in version of panel (**a**) is included in Supplementary Information (Fig. S3).

Figure 4a shows the magnetization dynamics based on the extended M3TM for different thicknesses of Ni thin film. Results show that the film thickness has a minimal influence on timescales of the demagnetization and recovery. However, it changes the demagnetization ratio (the dip on the magnetization curve). The effect of thickness on the magnetization dip is shown in Fig. 4b for different L_z_ values 10– 50 Å. In Fig. 2, due to the change in the DOS_F_ at sub-5 nm (50 Å) thickness regime, the electron–phonon coupling, and Sommerfeld coefficient change considerably with increasing film thickness. In Figs. 2 and 4a,b, the dips have the same positions, however, their amplitudes are different. This indicates that the change in the rate (G_ep_) triggers a stronger magnetization loss/recovery without altering the spin wave emission spectra.

Figure 4c,d show the dependence of T_e_ and T_p_ maxima on the film thickness, respectively. The modulation of the electron temperature maxima with increasing the film thickness is similar to the behavior of G_ep_ (Fig. 2) and demagnetization dip (Fig. 4b). The electron temperature and the magnetization dip change sharply for certain values of thickness, e.g., 22 to 22.5 Å (or 25.5 to 26 Å, 29 to 29.5 Å, 32.5 to 33 Å, 36 to 36.6 Å, 39.5 to 40 Å, 43 to 43.5 Å, 46.5 to 46 Å). The electron temperature can go up to 3300 K depending on L_z_. The extreme sensitivity of electron temperature (i.e. 3300 to 2620 K) to the thickness L_z_ (22 to 22.5 Å) shows that piezoelectric modulation can be a viable method for controlling “hot electrons”. The fact that electron temperature exceeds the Curie temperature does not prevent the nanomagnets from recovering magnetization. Due to increasing electron temperature, chemical potential gradient causes charge currents within metallic nanomagnets (see µ as a function of T_e_ in the Supplementary Fig. S2a). Still, since the highest electron temperature never exceeds 3300 K, the chemical potential difference is less than 1%.

We also investigate the sensitivity of our results to the size of the potential well and include them in the Supplementary Information (Figs. S4–S7), the finite size of the well does not change the qualitative predictions of our model. Particularly, independent of the quantum well depth, the quantum confinement effect reduces G_ep_, and consequently affects the transient magnetization behavior as well as the electron and phonon temperatures. This also affects the laser energy needed for manipulation of the magnetization, as discussed extensively in the previous part.

## Discussion

### Energy transfer from a fs laser pulse to the lattice and magnon system

Considering the lack of spin coupling, the fs laser pulse manipulates the magnetization without excess energy concentration in the lattice, resulting in the minimal change of phonon temperature. If we assume that the heat capacity of the nm thick Ni film is equal to the bulk value, with the Gaussian laser pulse fluence of I_0_ = 28 mJ m^−2^ and the spot size of 100 µm, the temperature change of a 2 nm-thick Ni film with the density of 8900 kg m^−3^, is calculated as E_laser_ = 2.184 × 10^–10^ J = M × c × ∆T · M (mass) = ρ_Ni_ ·V = 1.4 × 10^–13^ kg and the specific heat capacity of Ni (c) is 440 J (kg °C)^−1^. As a result, the temperature change in the thin film is ∆T = 3.545 °C. The laser fluence ranges for ultrafast magnetization switching of metallic magnetic thin films vary from 1 to 14 mJ cm^−2^ (10–140 J m^−2^) in the literature^12,44,45^. Even if we consider an upper limit of 10 J m^−2^ as incoming laser energy, the thin film temperature change would be ∆T = 1276 °C, which is still below the melting point of the Ni (1455 °C). Therefore, as long as the fluence is low, there is no significant thermal drift in the metal.

After equilibration with the electron bath all the laser pulse energy goes to the phonon bath. So the energy absorbed by the phonon is equal to E_p_ = C_p_· ∆T_p_. The maximum phonon temperature change is for the film with the thickness around 35 Å, which is around 0.022 K. So the energy absorbed by the phonons is simply E_p_ = (2.33 × 10^6^ J m^−3^ K) × (π/4 × (100 × 10^–6^ m) ^2^) × (3.5 × 10^–9^ m) × (0.022 K) = 1.4 × 10^–12^ J. From thermodynamic standpoint, phonon energy change (heat) is the difference between the injected energy into the system from laser pulse and the energy change in the spins (work done). The efficiency of work done by laser pulse on spins is η = 1 − Q_phonon_/Q_laser_. The absorbed energy for increasing the lattice temperature is Q_phonon_ = 1.4 × 10^–12^ J and the laser energy is 2.184 × 10^–10^ J. The efficiency is η = 99.36%. In Supplementary Information, we consider a condition where the spin-electron, and spin-phonon scattering are not neglected. Since a part of the laser energy is lost due to the scattering phenomena, the laser energy needed to manipulate and recover the magnetization increases. Considering the laser fluence of 35 mJ m^−2^ (Q_laser_ = 2.73 × 10^–10^ J), the increase in the phonon temperature maxima for 35 Å film does not considerably change, which is shown in Figure S8b in the Supplementary Information. However, due to the excess laser energy needed for recovery of the magnetization, the work done by the laser on the spins increases which increases the efficiency to η = 99.48%.

In summary, we investigated the all-optical magnetization dynamics in the quantum confined magnetic Ni ultrathin films. Our theoretical model shows that due to the quantum confinement in the films with the thicknesses of a few tens of angstrom, the electron–phonon coupling coefficient (G_ep_) in the M3TM is highly sensitive to the film thickness and could not be considered constant. This effect changes the amount of magnetization drop after interaction with the fs laser pulse, but not the timescales of the magnetization. In addition, we show that our qualitative predictions of magnetization dynamics in the quantum-confined ultrathin magnetic films are not sensitive to the size of the quantum well. We show that, laser-induced magnetization dynamics could drive ultrafast exchange-driven magnetization oscillations^46^. Furthermore, the quantum confinement effect decreases the lattice temperature change due to the lower laser fluences needed for magnetization control in Ni ultrathin film. Thus, the energy efficiency of exciting spin waves with lasers could be enhanced^47^.

Our study shows that the choice of the film thickness in the angstrom regime could help modulation of magnetization with around three orders of magnitude lower laser fluences compared to the reported experimental values. We also show that the energy transfer rate from the laser pulse to the lattice is so low that the lattice temperature stays far below both Curie temperature and the melting point of the Ni. The quantization of energy levels perpendicular to the film due to the thin film’s electron confinement leads to the oscillatory dependence of many properties on the film thickness due to quantum size effects. Despite the small oscillations as the result of quantum confinement, the effect is measurable by various experimental means such as magneto-thermoelectric measurements^48^, Kelvin probe force microscopy^49^, Hall-bar magnetoresistance measurement^50^, and scanning tunneling spectroscopy (STS) technique^51^. These methods are reported to measure the quantum size effect in different metallic and also more complex thin films.

In addition, this study suggests the researchers to expand further the understanding the nature of laser-matter interaction under the effect of quantum confinement on the laser-induced dynamics including the electron temperature profile affected by optical reflectivity and optical extinction in nanolayers and nanostructures.

## Methods

### Quantum confinement effects on DOS, G_ep_, and γ

Our physical system, which consists of a ferromagnetic ultrathin metal illuminated by an ultrashort (femtosecond) laser pulse, can be microscopically described by a generic Hamiltonian for interacting electron and phonon baths that reads,

5 $${\text{H}} = {\text{H}}_{{\text{e}}} + {\text{H}}_{{\text{p}}} + {\text{H}}_{{{\text{ep}}}}$$

where H_e_ and H_p_ are the Hamiltonians of free electrons and phonons, respectively. H_ep_ models the electron-scattering from the lattice, ignoring the spin flips. We remark that the baths are, in fact, not exactly free. The calculation of the electron–phonon coupling parameter G_ep_ in the two-temperature model proceeds by application of Fermi’s golden rule using H_ep_. In the Supplementary Information, we present a more extensive review on the derivation in Ref.^9^, which is based upon pioneering works^52^. It allows for including beyond free electron theory effects and arbitrary DOS.

We can write the temperature gradient between the baths such that

6 $${\mathrm{C}}_{\mathrm{e}}\frac{{\mathrm{dT}}_{\mathrm{e}}}{\mathrm{dt}}=-{\mathrm{G}}_{\mathrm{ep}}\left({\mathrm{T}}_{\mathrm{e}}-{\mathrm{T}}_{\mathrm{p}}\right)$$

electron–phonon coupling factor G_ep_ is given by

7 $${\text{G}}_{\text{ep}}={\uppi \hbar \uplambda }\langle {\upomega }^{2}\rangle {\text{g}}_{\text{F}}$$

where k_B_ is the Boltzmann constant, λ is the electron–phonon mass enhancement parameter^53^, and ⟨ω^2^⟩ is the second moment of the phonon^54^. At low temperatures, we can take C_e_ = γT_e_, where γ = π^2^k_B_^2^g_F_/3, according to the Sommerfeld expansion^55^. Numerical examination of C_e_ for different metals at higher temperatures, which is beyond the scope of the present contribution, can be found in the literature^1^. A similar equation can be obtained for the phonon bath. These equations can be changed to temperature rate equations using the corresponding specific heats. Finally, introducing the additional terms for the laser pulse absorption and heat diffusion, the two-temperature model^56^ can be developed.

The sensitivity of g_F_ to the variations of the thickness of the ultrathin film, L_z_, allows for additional control over the electron–phonon coupling G_ep_. We use the free electron model, while the electrons are confined in a potential well of size L_z_ and depth V_0_. This limits k_z_ to be always less than k_top_ = (2mV_0_)^1/2^/ħ. Quantization of k_z,_ according to^10^, reduces the finding temperature-dependent Fermi energy is a counting problem of electrons living on disks confined in the Fermi sphere with temperature-dependent radius.

8 $${\mathrm{K}}_{\mathrm{z}}{\mathrm{L}}_{\mathrm{z}}={\mathrm{n}}_{\mathrm{z}}\uppi -2{\mathrm{sin}}^{-1}\left(\frac{{\mathrm{K}}_{\mathrm{z}}}{{\mathrm{K}}_{\mathrm{top}}}\right)$$

Allowed k_z_ form a set k_z_ whose dimension is temperature dependent. The physical reason for the oscillations with L_z_ is due to the discreteness of the electronic wave number along the finite thickness direction, k_z_.

At zero temperature, the number of electrons can be determined by^10^.

9 $$\mathrm{N}=2\frac{{\mathrm{L}}_{\mathrm{x}}{\mathrm{L}}_{\mathrm{y}}}{4{\uppi }^{2}}{\sum }_{{\mathrm{k}}_{\mathrm{z}}\le {\mathrm{k}}_{\mathrm{F}}}\uppi \left({\mathrm{k}}_{\mathrm{F}}^{2}-{\mathrm{k}}_{\mathrm{z}}^{2}\right)$$

where L_x_, and L_y_ are the long, transverse sizes of the ultrathin film (L_z_ ≪ L_x_, L_y_). At finite temperatures, we should use $N=2{\sum }_{k}{f}_{k}$, where the Fermi–Dirac distribution would contain temperature-dependent Fermi energy µ(T) such that

10 $$\mathrm{N}=2\frac{{\mathrm{L}}_{\mathrm{x}}{\mathrm{L}}_{\mathrm{y}}}{4{\uppi }^{2}}{\sum }_{{\mathrm{k}}_{\mathrm{z}}\upepsilon {\mathrm{K}}_{\mathrm{z}}}\int {\mathrm{dk}}_{\mathrm{x}}{\mathrm{dk}}_{\mathrm{y}}\frac{1}{{\mathrm{e}}^{\left(\mathrm{E}\left(\mathrm{k}\right)-\upmu \left(\mathrm{T}\right)\right)/{\mathrm{k}}_{\mathrm{B}}\mathrm{T}}+1}$$

Using

11 $$\mathrm{E}\left(\mathrm{k}\right)=\frac{{\mathrm{\hbar }}^{2}}{2\mathrm{m}}\left({\mathrm{k}}_{\mathrm{x}}^{2}+{\mathrm{k}}_{\mathrm{y}}^{2}+{\mathrm{k}}_{\mathrm{z}}^{2}\right)$$

the integral can be evaluated analytically such that

12 $$\mathrm{n}=\frac{\mathrm{T}}{\uppi {\mathrm{L}}_{\mathrm{z}}}{\sum }_{{\mathrm{k}}_{\mathrm{z}}\upepsilon {\mathrm{K}}_{\mathrm{z}}}\mathrm{ln}\left[1+\mathrm{exp}\left(\frac{{\mathrm{k}}_{\upmu }^{2}-{\mathrm{k}}_{\mathrm{z}}^{2}}{\mathrm{T}}\right)\right]$$

where k_µ_ is the temperature-dependent Fermi wavenumber. We use scaled variables such that the length scale is a_B_, the temperature scale is T_B,_ and the energy scale is E_B_. This equation generalizes the zero temperature counting problem in Eq. (9), which determines the quantum confinement effect on DOS at Fermi energy to finite temperatures. In Supplementary Information, we describe a method in Ref.^10^ with which one could find how the L_z_ dependence of DOS at Fermi level changes with temperature, to be able to take into account this size effect for G_ep_. At the range of temperatures we are interested in, the temperature dependence of G_ep_ is negligibly different than the zero temperature results.

Assumptions that we considered in this manuscript are as the following:

1. The thin film is a thermally isolated and free-standing ultrathin layer of magnetic metal. So, due to its very low thickness, compared to the optical penetration depth of the laser pulse, the heat diffusion through the film thickness is neglected.
2. The spin-phonon and spin-electron coupling are neglected in our model. So, spin-related scattering events such as spin-phonon and spin-electron scattering is overlooked, which can be significant in the species with low spin–orbit coupling.
3. In the free electron model, we considered a rectangular electron potential well with the constant depth of V_z﻿_ = 10 eV.
4. Our model assumes that C_p_ is not dependent on the thickness and temperature in the considered time and thickness regime.
5. We neglect the heat conduction in the lattice, since in pure metal heat conduction is negligible compared to that of electron heat.
6. Due to the very short laser pulse and very low thickness of our films, we neglected non-thermal excitation after interaction with the laser pulse.

## Supplementary Information

Supplementary Information 1.

Supplementary Information 2.

## Data Availability

Supplementary information accompanies this paper along with the MATLAB codes containing our model calculations.
