# Supplementary material for: All optical control of magnetization in quantum confined ultrathin magnetic metals
Source: Sci Rep. 2021 Aug 5;11:15976. doi: 10.1038/s41598-021-95319-6 (PMC8342544; doi:10.1038/s41598-021-95319-6)
Supplement: Supplementary file 1 — Supplementary Information 1. [file 41598_2021_95319_MOESM1_ESM.pdf]

Supplementary Information for "All Optical Control of Magnetization in Quantum Confined Ultrathin Magnetic Metals"

Saeedeh Mokarian Zanjani[1*], Muhammad Tahir Naseem[2], Özgür Müstecaplıoğlu[2], Mehmet Cengiz Onbaşlı[1,3*]
[1]Graduate School of Materials Science and Engineering, Koç University, Sarıyer, 34450 Istanbul, Turkey.
[2] Department of Physics, Koç University, Sarıyer, 34450 Istanbul, Turkey.
[3] Department of Electrical and Electronics Engineering, Koç University, Sarıyer, 34450 Istanbul, Turkey.
[*] Corresponding Authors: monbasli@ku.edu.tr, szanjani16@ku.edu.tr

This supplementary information is designed in three main parts. In the first part, we present the complete derivation of the three temperature model, which we explained and used in the main manuscript. In the second part, we compare the thickness dependence of the chemical potential and the Fermi level electronic density of states for two different electron temperature (T_e). Furthermore, we investigate the sensitivity of our calculation results presented in the main paper to the potential well depth (V_z). In the third part, we present the condition where we involve the spin-electron and spin-phonon coupling to justify the ability of our model to explain the high efficiency of energy transfer to the spin system using quantum-confinement in the all-optical magnetization manipulation. In the rest of this supplementary document we investigate the effect of pulse width and pulse fluence on the magnetization dynamics and thickness dependent demagnetization ratio, electron and phonon temperatures. Next, we consider the hypothetical condition where only 20% of the laser fluence is absorbed by the Ni thin films as suggested in the literature. We compared the results of magnetization dynamics in Angstrom-thick Ni film with that of nm-thick non-quantum-confined Ni ultrathin film. Finally we investigate and compare the magnetization dynamics of the Angstrom-thick, and nm-thick Ni when illuminated with different pulse widths which spa from 50 fs to 5 ps.

Derivation of the microscopic three temperature model

Here we present a brief derivation of the microscopic three temperature model based on the pioneering works [1,2], including free electron theory. Then the effect of ultrathin film thickness on the electron density of state at the Fermi level, and eventually, the electron-phonon coupling coefficient is theoretically modeled.

This section is organized to present our theoretical derivation of the microscopic three temperature model. A ferromagnetic ultrathin metal illuminated by an ultrashort (femtosecond) laser pulse can be microscopically described by a generic Hamiltonian for interacting electron and phonon baths as the following:

$$H = H_e + H_p + H_{ep} \qquad \text{(S1)}$$

where H_e and H_p are the Hamiltonians of free electrons and phonons, respectively. H_{ep} models the electron-scattering from the lattice, ignoring the spin flips. We remark that the baths are, in fact, not exactly free. The interactions in the same subsystems H_{ee} and H_{pp} lead to fast equilibration and hence, they are dropped after instant thermalization assumptions. Their effects are implicit in the dynamics; we will not express them here explicitly. Ignoring the spin degree of freedom of the electrons, non-interacting gas of them has the Hamiltonian

$$H_e = \sum_k E(\mathbf{k})\mathbf{c}_{\mathbf{k}}^{\dagger}\mathbf{c}_{\mathbf{k}} \quad \text{(S2)}$$

Here $c_{\mathbf{k}}$ ($c^{\dagger}_{\mathbf{k}}$) annihilates (generates) an electron with excitation energy E(**k**) in Bloch state |**k**⟩. Initially, the gas in metal is in equilibrium at an ambient temperature T_0. After the ultrashort pulse excitation, this gas can quickly reach a new temperature due to (screened) Coulomb interaction H_{ee}.

An ensemble of non-interacting quantum harmonic oscillators describes the bath of lattice vibrations, phonons, with the Hamiltonian

$$H_p = \sum_q \hbar\omega_q(\mathbf{a}_{\mathbf{q}}^{\dagger}\mathbf{a}_{\mathbf{q}} + 1/2) \quad \text{(S3)}$$

Here the operators $a^{\dagger}_{\mathbf{q}}$ and $a_{\mathbf{q}}$ generate and annihilate phonons with quasi-momentum **q**, respectively. Phonons obey the Bose-Einstein statistics. Einstein model is the simplest choice to describe them, while Debye model is also possible.

Fröchlich interaction describes the scattering of spinless electrons from the lattice as

$$H_{ep} = \sum_{kk'q} V_{kk'q}\mathbf{c}_{\mathbf{k}}^{\dagger}\mathbf{c}_{\mathbf{k}'}\,(\mathbf{a}_{\mathbf{q}}^{\dagger}+\mathbf{a}_{\mathbf{q}}) \quad \text{(S4)}$$

where we denote the matrix element of the scattering process with $V_{\mathbf{kk'q}}$.

The calculation of electron-phonon coupling parameter G_{ep} in the two-temperature model proceeds applying of the Fermi's golden rule using H_{ep}. We will present a brief review of the derivation in Ref. [1], which is based upon pioneering works [2] , and allows for including beyond free electron theory effects and arbitrary DOS. Accordingly, we write the energy transfer rate from electrons to the lattice E_e as

$$\frac{\partial E_e}{\partial t} = \frac{4\pi}{\hbar}\sum_{kk'} \hbar\omega_q |V_{kk'}|^2 S(k,k')\delta(E(k) - E(k') + \hbar\omega_q) \quad \text{(S5)}$$

Here,

$$S(k,k') = (f_k - f_{k'})n_q - f_{k'}(1 - f_k) \quad \text{(S6)}$$

is the thermal factor, that depends on the Fermi-Dirac ($f_{\mathbf{k}}$) and Bose-Einstein ($n_{\mathbf{q}}$) distribution functions of the electron and phonon baths in their "local" thermal equilibrium states [1]. Explicitly they are defined as $f_{\mathbf{k}} = 1/(1 + \exp{(E(k) - \mu)/k_B T_e})$ and $n_{\mathbf{q}} = 1/(\exp{(\hbar\omega_q/k_B T_p} - 1)$. Near room temperature, after the conversion of the k summation to continuum energy integrals, we can write

$$\frac{\partial E_e}{\partial t} = 2\pi g_F \int d\Omega \alpha^2 F(\Omega)(\hbar\Omega)^2\left[n(\hbar\Omega, T_p) - n(\hbar\Omega, T_e)\right] \quad \text{(S7)}$$

with g_F denoting the electronic DOS at Fermi level. The limitation of the DOS to the Fermi level value assumes that only those electrons near the Fermi energy could contribute to the scattering process from the lattice vibrations around room temperature. The term $\alpha^2 F(\mathbf{\Omega})$ is the Eliashberg spectral function [3]. If we assume temperatures are below Debye temperatures but higher than phonon mode energy $\hbar\mathbf{\Omega} \ll k_B T_p$, $k_B T_e$, then we can replace the population distributions with a temperature gradient between the baths such that

$$C_e \frac{dT_e}{dt} = -G_{ep}(T_e - T_p) \quad \text{(S8)}$$

where we used $dE_e = C_e dT_e$. Electron-phonon coupling factor G_{ep} is given by

$$G_{ep} = \pi\hbar\lambda\langle\omega^2\rangle g_F \quad \text{(S9)}$$

where k_B is the Boltzmann constant, λ is the electron-phonon mass enhancement parameter [4], and $\langle\omega^2\rangle$ is the second moment of the phonon [3]. At low temperatures, we can take $C_e = \gamma T_e$, where $\gamma = \pi^2 k_B^2 g_F/3$, according to the Sommerfeld expansion [5]. Numerical examination of C_e for different metals at higher temperatures, which is beyond the scope of the present contribution, can be found in the literature [6].

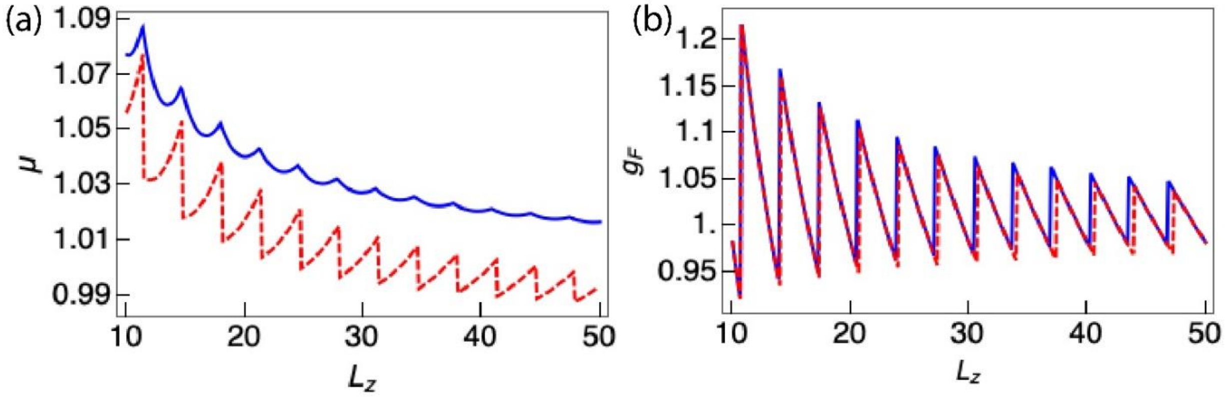

Figure S1. (**a**) Chemical potential (finite temperature Fermi energy) μ and (**b**) Density of states (DOS) at the Fermi energy as a function of ultrathinfilm thickness Lz (in units of A). Both μ and g_F are dimensionless, normalized with their corresponding bulk values. Solid blue lines are the zero temperature results. Dashed red lines are for the electronic temperature $T_e = 5 \times 10^{-3} T_B$, where $T_B = E_B/k_B$ being the Bohr temperature corresponding to the Bohr energy E_B = 13.6 eV. Depth of the metallic confinement potential is taken to be V_z = 10 eV. Electronic density is used as $n = 3/4\pi r_s^3$ with $r_s = 4a_B$, and a_B being the Bohr radius.

Dependence of DOS_F on temperature

In this section, we describe a theoretical method with which the dependence of DOS at Fermi level changes with temperature, to be able to take into account this size effect for G_{ep}.

Our numerical method starts with taking k_z for $n_z = 1$ in Eq. (S4) as a guess set of K_z to evaluate k_μ by solving Eq. (S8) in the main manuscript. We then compare k_z with k_μ; if $k_z < k_\mu$, then we increase the size of K_z by increasing n_z one more. The iteration repeats till the largest k_z in K_z is more than calculated k_μ. The results for corresponding chemical potential μ (or temperature-dependent Fermi energy) and DOS at Fermi energy are plotted in figure S2. Both μ and g_F are dimensionless, normalized with their corresponding bulk values. Generalizing the methods in Ref. [7] to this temperature-dependent Fermi energy case, we found how the L_z dependence of DOS at Fermi level changes with temperature, to be able to take into account this size effect for G_{ep}.

In figure S2, the chemical potential change with electron temperature, and Fermi level density of state, are shown. In addition, the electron phonon coupling coefficient is shown in figure S2(c). Both DOS_F and G_{ep} are independent of the electron temperature at the temperature ranges applicable in our calculations (where maximum electron temperature does not exceed 2000 K).

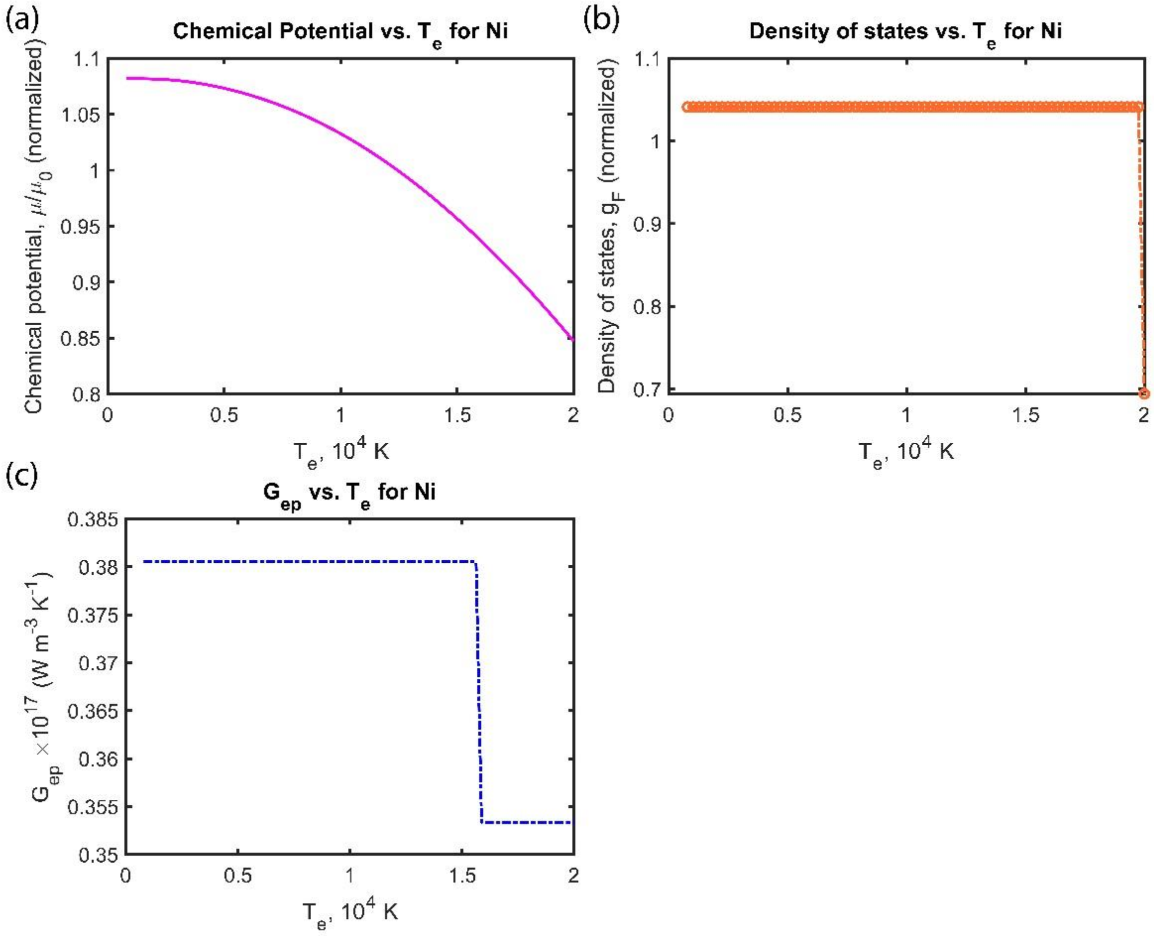

Figure S2. The dependence of the Fermi level (**a**) chemical potential (m), (**b**) density of states, and (**c**) electron phonon coupling (G_{ep}) on the electron temperature (T_e).

The effect of substrate (especially if it is non-magnetic) might be negligible in changing of the magnetization order. However, it might influence the contributing electron and phonon density and so G_{ep}. Nevertheless, assuming an isolated thin film is reasonable considering the previous studies [8,9].

In figure 4 of the main manuscript, we showed the magnetization dynamics of the Ni ultrathin film for different film thicknesses. A zoomed in version of the magnetization dynamics at a smaller time ranges are shown in figure S3.

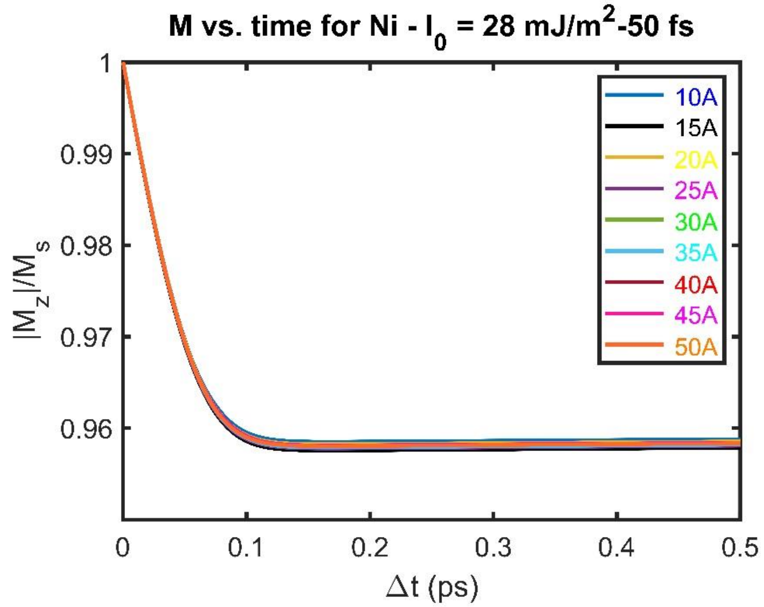

Figure S3. Magnetization dynamics of the Ni ultrathin film in 300 fs after interaction with the laser pulse.

Sensitivity of quantum confinement effect of magnetic ultrathin films to the potential well depth (V_z)

In the main manuscript, we reported our calculation results of quantum confinement effect in figure, 2 and 4, using the potential well depth of V_z= 10 eV. In this section of SI document, we present the sensitivity of our results to the size of the potential well. Practically, the V_z is an infinite value, however, in the FEM calculations, a finite value is given to it. We consider three extra cases of V_z=5 eV, 15 eV, and 20 eV.

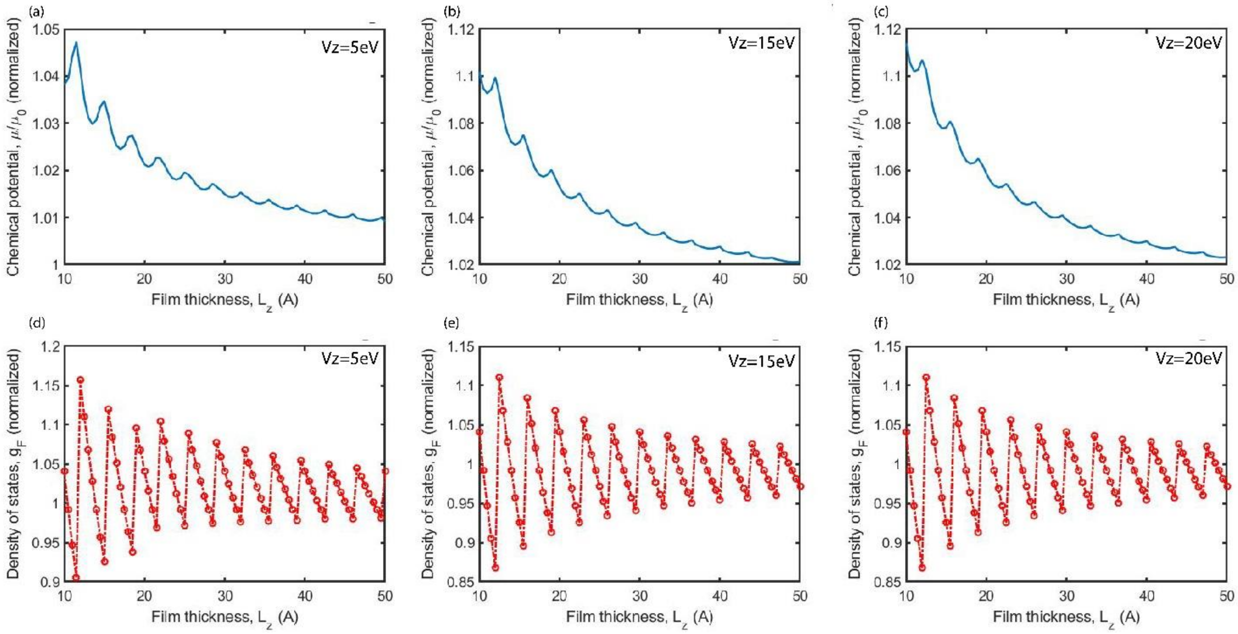

Figure S4. Thickness dependent (**a**) chemical potential of the quantum confined Ni ultrathin film with quantum well depth of V_z=5 eV, (**b**) 15 eV, (**c**) 20 eV, (**d**) Fermi level electron density of states for V_z= 5 eV (**e**) 15 eV (**f**) 20 eV.

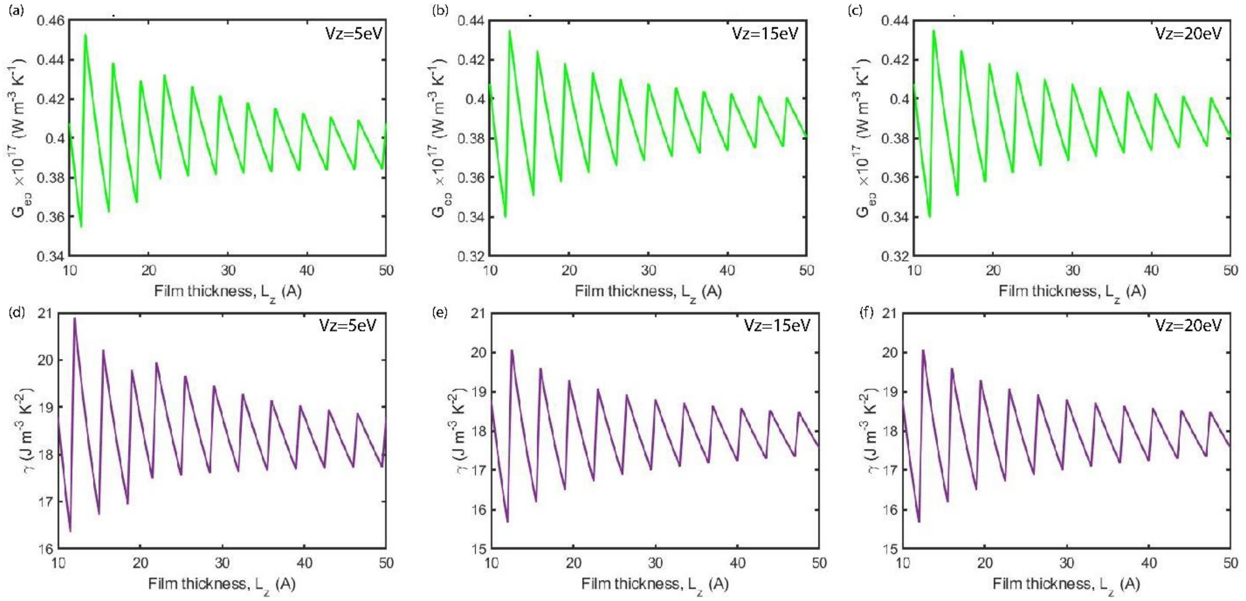

Figure S5. Thickness dependent (**a**) electron-phonon coupling coefficient the quantum confined Ni ultrathin film with quantum well depth of V_z=5 eV, (**b**) 15 eV, (**c**) 20 eV, (**d**) Sommerfeld coefficient for V_z= 5 eV (**e**) 15 eV (**f**) 20 eV.

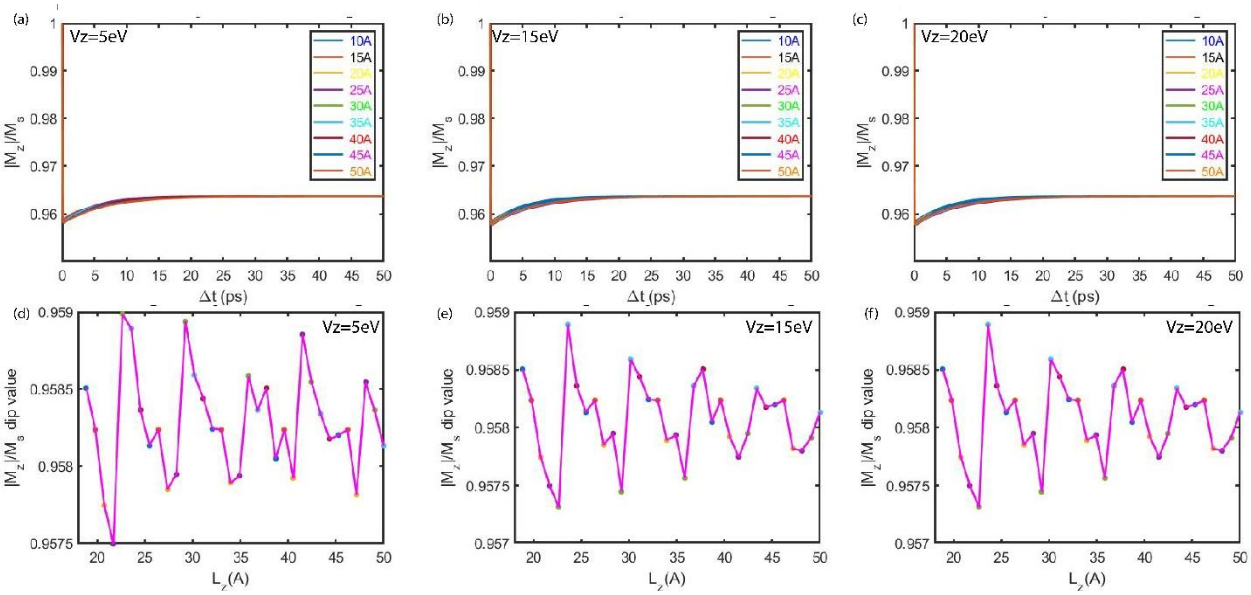

Figure S6. Thickness dependent (**a**) magnetization dynamics of the quantum confined Ni ultrathin film with quantum well depth of V_z=5 eV, (**b**) 15 eV, (**c**) 20 eV, (**d**) demagnetization dip for V_z= 5 eV (**e**) 15 eV (**f**) 20 eV.

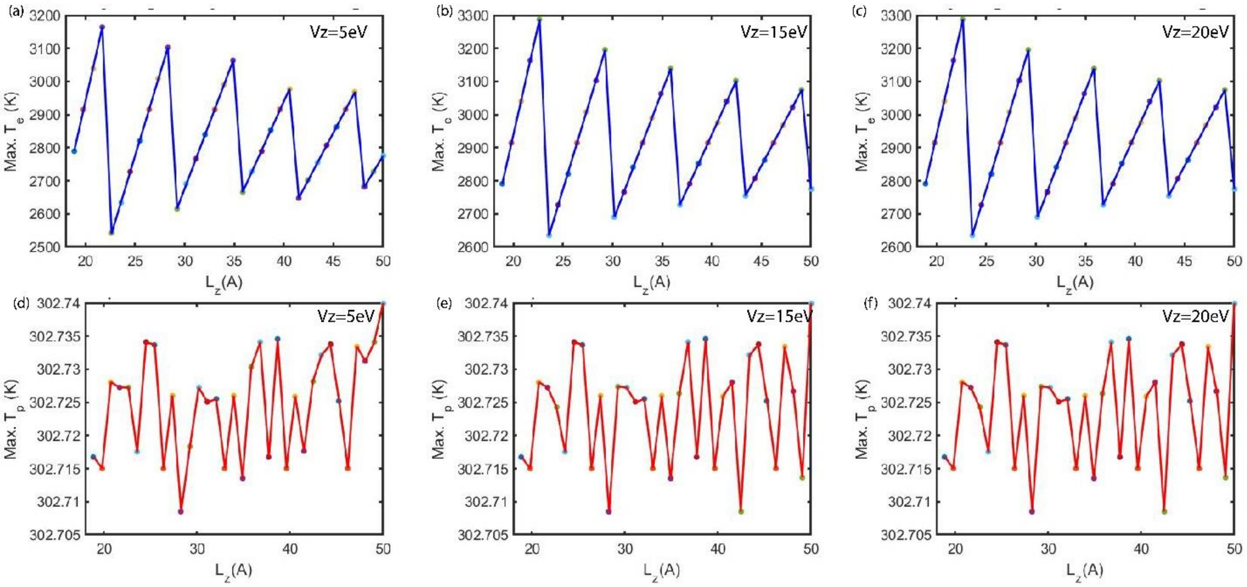

Figure S7. Thickness dependent (**a**) maximum electron temperature of the quantum confined Ni ultrathin film with quantum well depth of V_z=5 eV, (**b**) 15 eV, (**c**) 20 eV, (**d**) maximum phonon temperature for V_z= 5 eV (**e**) 15 eV (**f**) 20 eV.

The calculations results for the various potential well depths (V_z=5, 10, 15, and 20 eV) shows that only in case of sub-20 Å film thicknesses, V_z size might result in a few percent difference in the calculations. However, it does not influence the qualitative predictions of our model on decrease and oscillations in the electron density of states and electron-phonon coupling as the result of quantum confinement effect. In other words μ increases slightly (~ 3%), in sub-20 Å range, from V_z=5 eV to V_z=10 eV, but the oscillations are not considerable when V_z changes from 10 eV to 15 eV or 20 eV. Accordingly, g_F, G_{ep}, and γ oscillations decrease (~ 4%) changing from V_z=5 eV to V_z=10 eV, while remains unchanged for other V_z values. We also investigated the sensitivity of the figure 4 results to the size of V_z. Despite similar negligible variations for V_z= 5 eV in case of low film thicknesses, changing the depth of the potential well did not influence the conclusions of the quantum confinement effect on the oscillations of the magnetization dynamics as well as electron and phonon temperatures (figure S6-S7). Increasing the V_z from 5 eV, a small increase (~ 3%) in the maximum electron temperature and decrease in demagnetization dip was observed for sub-20 Å thin films. However, for other V_z values, the results remained unchanged. Since the decrease in the chemical potential and consequently the g_F, and G_{ep}, is averagely monotonic [7] (the amplitude of the oscillations does not exceed a few percent), the qualitative results of our calculations are consistent using different potential well depths.

M3TM model considering spin-phonon and spin-electron scattering

In this section, we calculated the effect of film thickness including the spin-phonon, and spin-electron scattering in the M3TM models shown in figure S8 (a). We used the following equations:

$$C_e \frac{dT_e}{dt} = -G_{ep}(T_e - T_p) - G_{es}(T_e - T_s) + P(t) \tag{1}$$

$$C_p \frac{dT_p}{dt} = -G_{ep}(T_p - T_e) - G_{ps}(T_p - T_s) \tag{2}$$

$$C_s \frac{dT_s}{dt} = -G_{es}(T_s - T_e) - G_{ps}(T_s - T_p) \tag{3}$$

$$\frac{dm}{dt} = Rm\frac{T_p}{T_C}\left(1 - m\coth\left(m\frac{T_C}{T_e}\right)\right) \quad (4)$$

where C_s is the spin heat capacity, G_{es}, and G_{ps} are the electron-spin and phonon-spin coupling coefficients, respectively. T_s represents the spin temperature. Note that both G_{ep} and R change with the film thickness, as mentioned in the manuscript. if we heuristically assume that G_{es} and G_{ps} have similar size dependence as with G_{ep}, through their dependence to DOS_F, we find that our results with constant G_{ep}, and G_{es} do not change significantly. We therefore intuitively expect that the size dependence of G_{es} and G_{ps} are negligible on T_e and T_p dynamics, which determine the magnetization evolution.

According to the figure S8(a), due to the energy loss to the spin-phonon scattering phenomena, the laser fluence needed to recover the magnetization is larger compared to the condition where the spin-lattice scattering is neglected (I_0=35 $mJ\cdot m^{-2}$). In addition, the magnetization drop time is slightly longer, while the recovered magnetization ratio is slightly lower as the result of spin scattering. Figure S8 (b) shows the thickness dependence of the lattice temperature. According to this figure, the maximum lattice temperature increases due to the energy exchange between spin and phonon baths. However, maximum T_p does not exceed 305.976 K, which is still well below the Curie temperature of Ni.

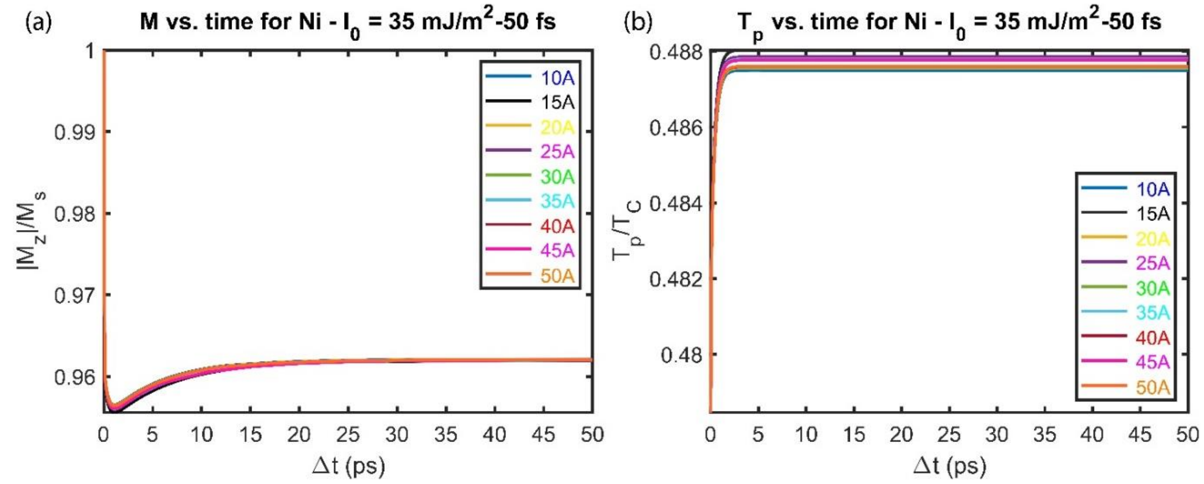

Figure S8. Effect of Ni film thickness on (**a**) magnetization dynamics for L_z = 10 Å, 15 Å , 20 Å, 25 Å, 30 Å, 35 Å, 40 Å, 45 Å, and 50 Å thick Ni films, (**b**) T_p/T_C, illuminated with I_0 = 35 mJ·m-2 Gaussian single laser pulse.

In the previous studies, the easy axis of the Ni changes from perpendicular to in-plane when the film thickness changes from around 12-14 Å to 24 Å [10,11]. The size-dependent change in the magnetic anisotropy is due to shape anisotropy (dominant anisotropy in the thicker films that dictate the in-plane easy axis), which is completely geometry dependent. In our magnetization dynamics model, the "m" stands for the normalized magnetization vector magnitude ($|M_z/M_s|$). After interaction with the low-fluence fs laser pulse, the magnetization vector orientation might change but not switch completely. The motion is described as precession, canting, or maybe toggle from an initial stable orientation, which we assume to be m_0=1, regardless of the direction. The magnetization magnitude, which changes due to spin reorientation, does not influence the temperature dynamics in the 2TM or M3TM. The easy axis and magnetic anisotropy effects are

studied using LLG equations where the magnetization is a vector, which is beyond our paper's scope [12-15].

Effect of the femtosecond laser pulse width on the magnetization dynamics of the quantum confined ultrathin Ni film

According to the laser pulse power distribution, $P(t) = \frac{P_0}{\sqrt{2\pi}} \exp\left(-\frac{1}{2}\left(\frac{t}{t_0}\right)^2\right)$ the wider pulse leads to smaller power/energy injected to the thin film ($P_0 = \frac{I_0}{d.t_0}$, and I_0 and t_0 are the laser pulse fluence in $J{\cdot}m^{-2}$, pulse width (fs), respectively). It leads to increase in the time of the demagnetization and also electron temperature equilibration. Moreover, for wider pulses such as 200 fs and 500 fs, the injected energy is small such that the quenched magnetization does not recover back and stays constant, and the demagnetization ratio is smaller compared to the lower pulse widths. Electron temperature rises to the lower amounts in case of wide laser pulses, due to the lower laser energy injection and similar to the magnetization quenching time, the electron equilibration time increases by increasing the pulse width. This effect is also reflected in the maximum electron temperature, which is considerably smaller for 200 fs and 500 fs pulse width, compared to 50 fs. In addition, figures S9-S11 show that for each specific pulse width, the behavior of the magnetization dip oscillation and maximum T_e and T_p are almost similar. In conclusion, increasing the pulse width decreases the laser power injected to the thin film which leads to increase in the demagnetization time as well as electron equilibration time. Choosing very long pulse durations, increases the laser fluence needed for recovery of the magnetization after quenching, which is not favorable for the scope of our manuscript.

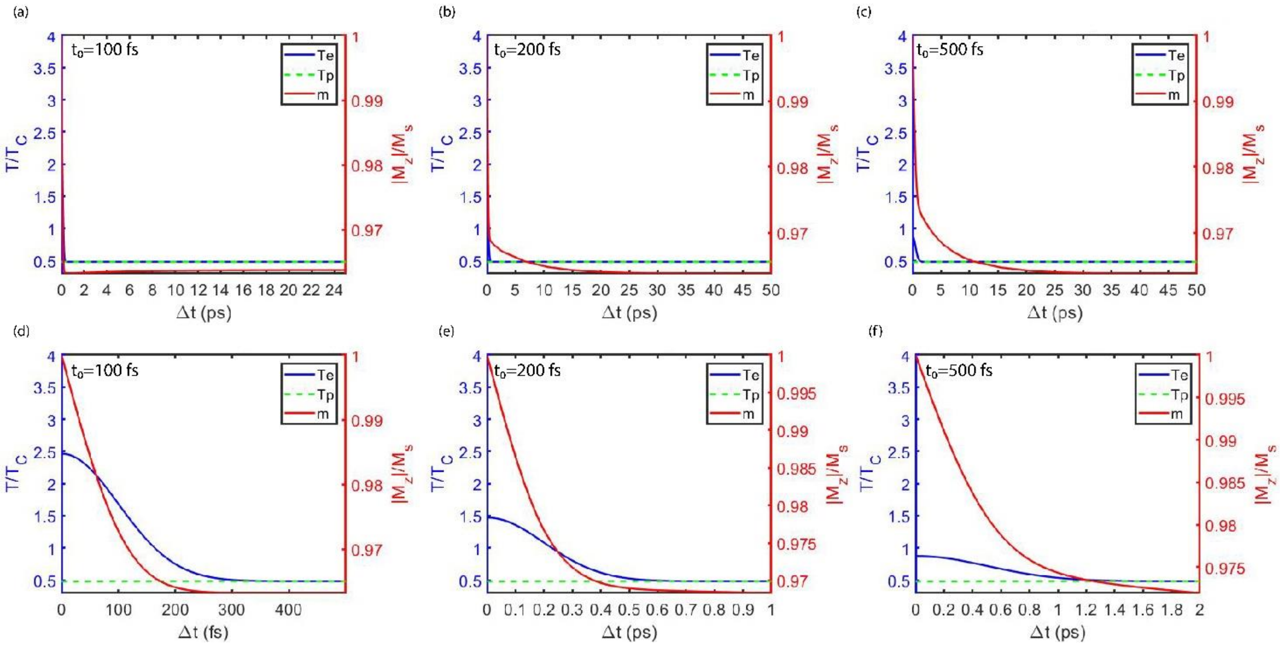

Figure S9. Magnetization dynamics of 20Å Ni film under 28 $mJ{\cdot}m^{-2}$ laser pulse fluence of (**a**) 100 fs (**b**) 200 fs and (**c**) 500 fs pulse width. The figures (**d**)-(**f**) show the zoomed-in version of the (**a**)-(**c**) respectively.

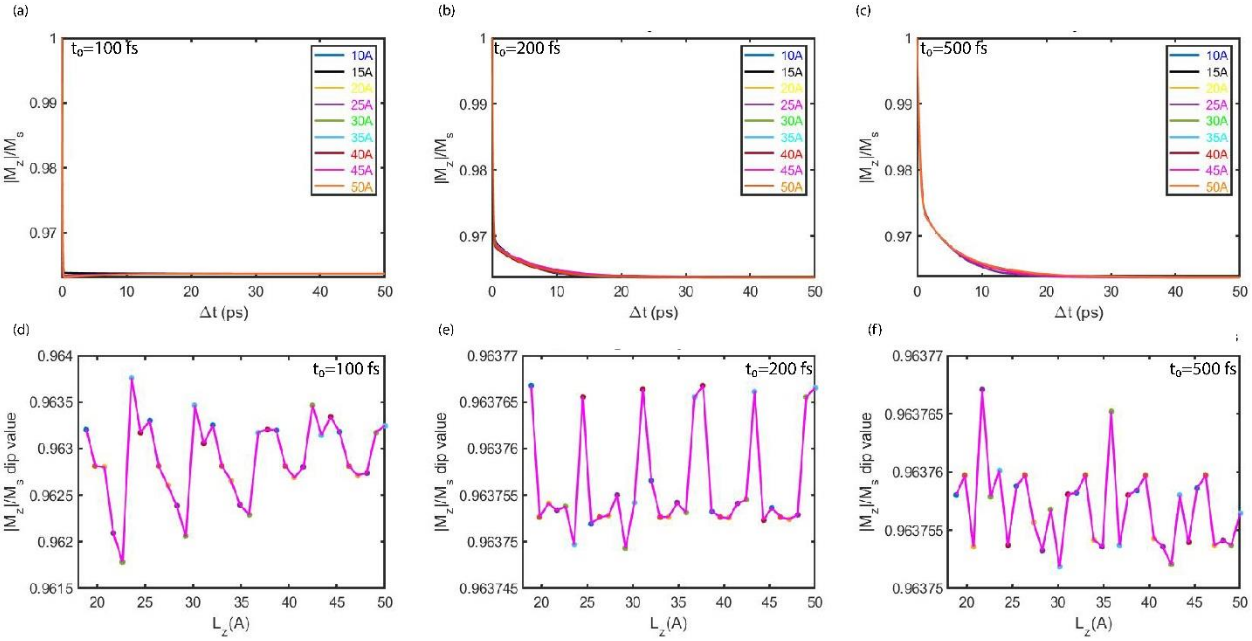

Figure S10. Thickness dependence of magnetization dynamics of Ni ultrathin film under 28 mJ·m^{-2} laser pulse fluence of (**a**) 100 fs (**b**) 200 fs and (**c**) 500 fs pulse width. The figures (**d**)-(**f**) show the magnetization dip values in (**a**)-(**c**) respectively.

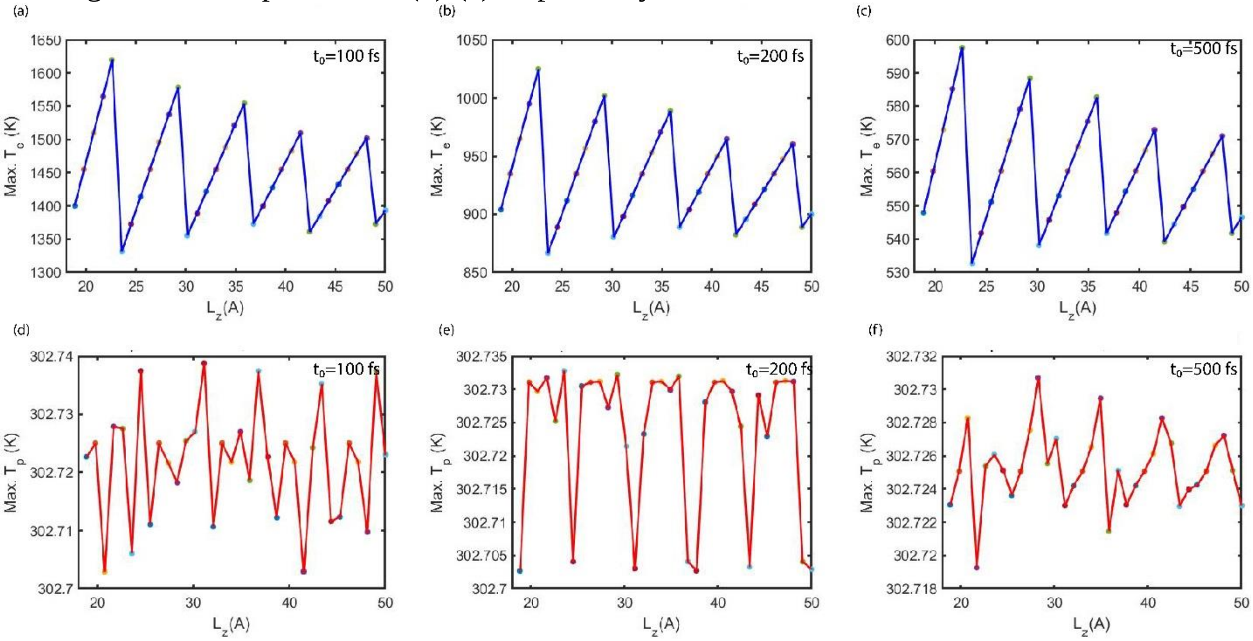

Figure S11. Thickness dependence of maximum electron temperaturein Ni ultrathin film under 28 mJ·m^{-2} laser pulse fluence of (**a**) 100 fs (**b**) 200 fs and (**c**) 500 fs pulse width. The figures (**d**)-(**f**) show the thickness dependence of maximum phonon temperature values for the pulse widths in (**a**)-(**c**) respectively.

Effect of laser pulse fluence on magnetization dynamics, and temporal electron and phonon temperatures

In the laser-driven all-optical switching experiments, not all the incoming laser energy is absorbed by the ultrathin Ni film; we investigate the case of Ni thin films with a few nm thickness, in which

only 20% of the incoming laser fluence is absorbed by the thin film and most of it transmits through the Ni sample. We have redone the calculations using incoming laser fluences of 2, 5, 7, and 10 mJ·cm^{-2} (ranges used in the experiments [16-18]) for both nm-thick Ni film using M3TM model of Koopmans, and quantum confined Ni thin film. The results are shown in Figure S12.

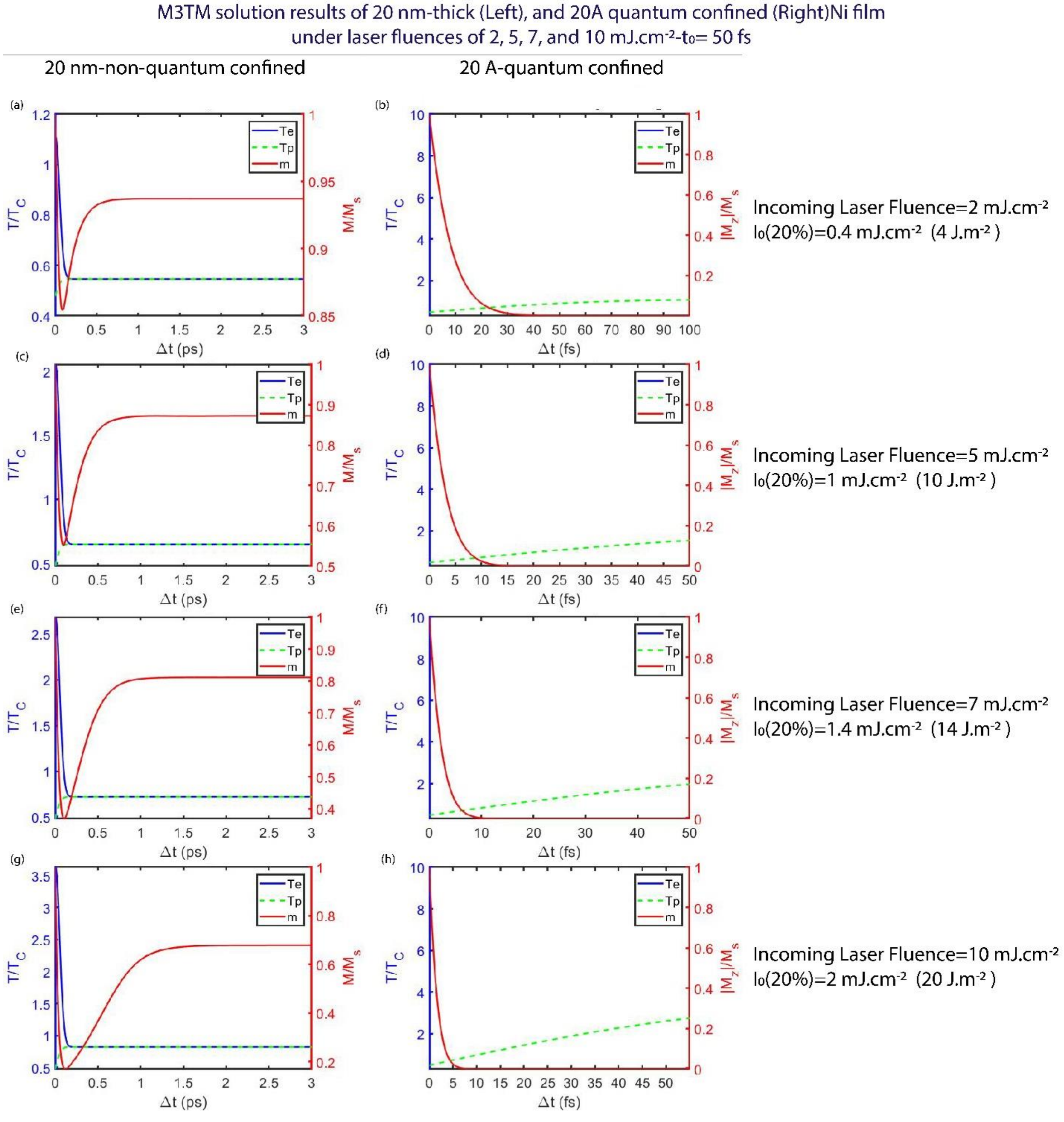

Figure S12: M3TM solution results of 20 nm-thick (**a**, **c**, **e**, **g**), and 20 Å quantum-confined (**b**, **d**, **f**, **h**) Ni film when it absorbs 20% of the incoming laser fluences. Incident laser fluences are (**a**, **b**) 0.4 mJ·cm^{-2}, (**c**, **d**) 1 mJ·cm^{-2}, (**e**, **f**) 1.4 mJ·cm^{-2}, and (**g**, **h**) 2 mJ·cm^{-2}. Note that the plots of the quantum confined film (right) are a zoomed-in version (to show the complete thermal

demagnetization and T_p exceeding T_C), before the equilibration of electron and phonon temperatures.

The results of Figure S12 show that using the same experimental parameters for solving M3TM, results in magnetization drop in sub-200 fs and recovery to near 65%-95% of its initial stage in 1-2 ps (depending on the laser fluence). The plots on the right side of Figure S12 shows that, despite the nm-thick Ni film, using similar laser fluences, the lattice temperature exceeds the Curie temperature in the quantum confined 20 Å Ni film and results in complete thermal demagnetization, even before the electron-phonon equilibration (in case the material is not evaporated).

Effect of laser pulse width on the magnetization dynamics, and temporal electron and phonon temperatures

The effect of the laser pulse duration (width) appears in the incident laser power to the thin film. According to the $P(t) = \frac{P_0}{\sqrt{2\pi}} \exp\left(\frac{-1}{2}\left(\frac{t}{t_0}\right)^2\right)$ formula, by increasing the pulse duration, t0, the injected laser energy to the thin film decreases which leads to a lower rise in T_e, when the pulse width is higher.

We have shown and compared the results of M3TM both for nm-thick (experimental parameters [8,9]) and quantum confined Ni film for different pulse widths in fs and ps range, shown in Figures S13 and S14 of this supporting information, respectively. We show that using the pulse width in sub 500 fs timescales, the sudden rise in the electron temperature is inevitable due to the excess energy concentration and low heat capacity of the electron. The conclusions from Figures S13 and S14 are as follows:

1) In the case of illumination with the pulse widths in the picosecond regime, the rise in the electron temperature happens more smoothly.

2) Due to the lower energy injection in the case of wider pulses, the magnetization recovery is suppressed, and it stabilizes after magnetization quenching.

3) The timescales of the magnetization quenching and recovery increase in the case of wider pulses, which is undesirable for ultrafast manipulation of magnetization.

4) Electron temperature equilibration time increases with increasing the pulse width/duration.

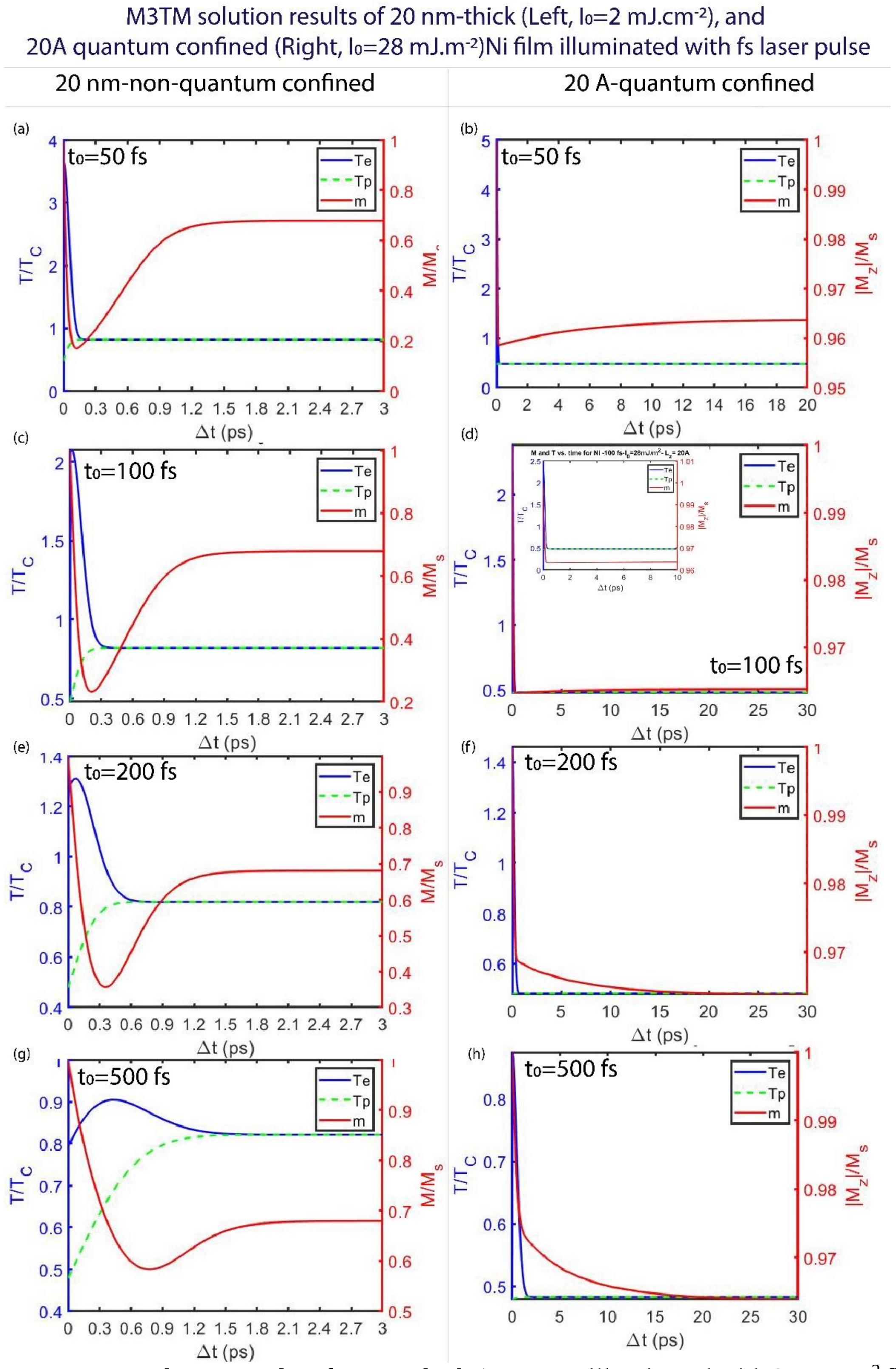

Figure S13: M3TM solution results of 20 nm-thick (**a**, **c**, **e**, **g**: illuminated with 2 mJ·cm^{-2} fluence), and 20 Å quantum-confined (**b**, **d**, **f**, **h**: illuminated with 2 mJ·cm^{-2} fluence) Ni film using a pulse width of (**a**, **b**) 50 fs, (**c**, **d**) 100 fs, (**e**, **f**) 200 fs, and (**g**, **h**) 500 fs.

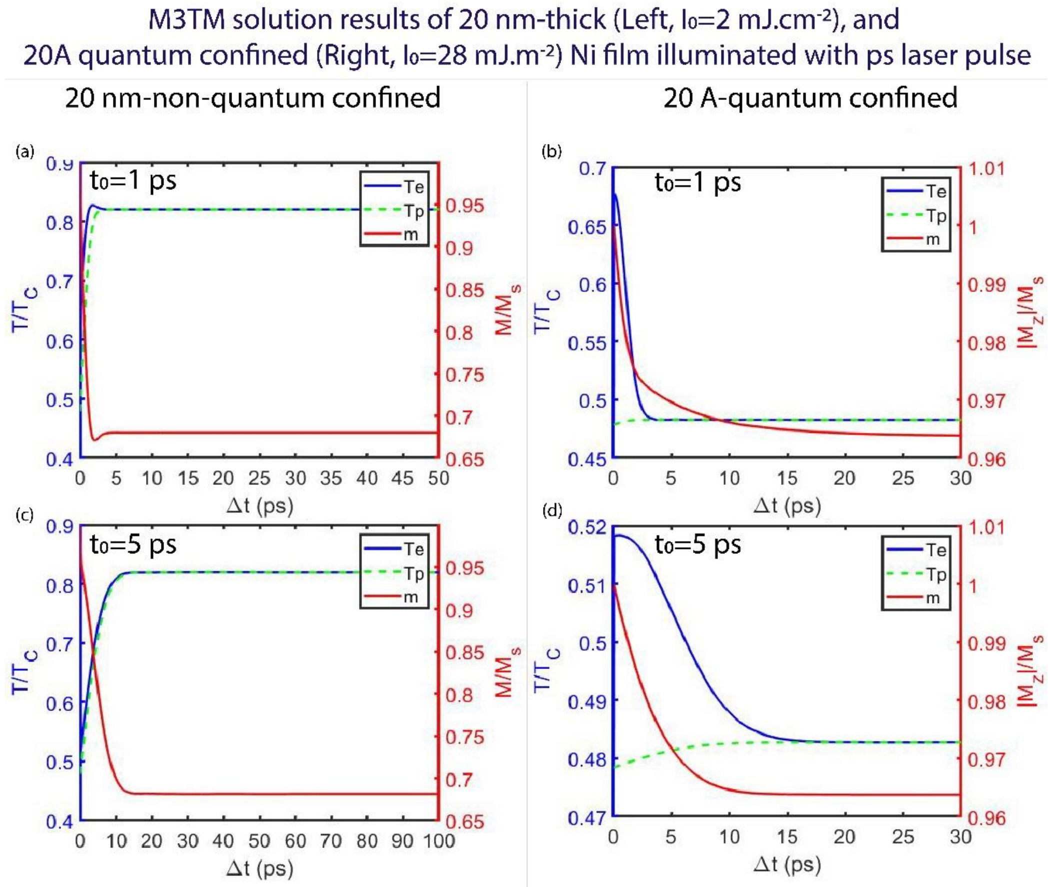

Figure S14: M3TM solution results of 20 nm-thick (**a**, **c**, **e**, **g**: illuminated with 2 mJ·cm^{-2} fluence), and 20 Å quantum-confined (**b**, **d**, **f**, **h**: illuminated with 2 mJ·cm^{-2} fluence) Ni film using a pulse width of (**a**, **b**) 1 ps, and (**c**, **d**) 5 ps.

References

1 Allen, P. B. Theory of thermal relaxation of electrons in metals. *Physical review letters* **59**, 1460 (1987).

2 Kaganov, M., Lifshitz, E. & Tanatarov, L. Relaxation between electrons and the crystalline lattice. *Soviet Physics-JETP* **4**, 173-178 (1957).

3 McMillan, W. Transition temperature of strong-coupled superconductors. *Physical Review* **167**, 331 (1968).

4 Grimvall, G. The electron-phonon interaction in metals. (1981).

5 Ashcroft, N. W. & Mermin, N. D. (New York: Holt, Rinehart and Winston, 1976).

6 Lin, Z., Zhigilei, L. V. & Celli, V. Electron-phonon coupling and electron heat capacity of metals under conditions of strong electron-phonon nonequilibrium. *Physical Review B* **77**, 075133 (2008).

7 Rogers III, J., Cutler, P., Feuchtwang, T. & Lucas, A. Quantum size effects in the fermi energy and electronic density of states in a finite square well thin film model. *Surface Science* **181**, 436-456 (1987).

8 Koopmans, B. *et al.* Explaining the paradoxical diversity of ultrafast laser-induced demagnetization. *Nature materials* **9**, 259-265 (2010).

9 Beaurepaire, E., Merle, J.-C., Daunois, A. & Bigot, J.-Y. Ultrafast spin dynamics in ferromagnetic nickel. *Physical review letters* **76**, 4250 (1996).

10 Schulz, B. & Baberschke, K. Crossover from in-plane to perpendicular magnetization in ultrathin Ni/Cu (001) films. *Physical Review B* **50**, 13467 (1994).

11 Parlak, U., Akoz, M., Tokdemir Ozturk, S. & Erkovan, M. Thickness dependent magnetic properties of polycrystalline nickel thin films. *Acta Physica Polonica A* **127**, 995-997 (2015).

12 Corrêa, M. A. & Bohn, F. Manipulating the magnetic anisotropy and magnetization dynamics by stress: Numerical calculation and experiment. *Journal of Magnetism and Magnetic Materials* **453**, 30-35 (2018).

13 d'Aquino, M. Nonlinear magnetization dynamics in thin-films and nanoparticles. *http://wpage. unina. it/mdaquino/PhD_thesis/main/main. html* (2004).

14 Giuffrida, C., Ragusa, C. & Repetto, M. Magnetization dynamics in metallic thin films by finite formulation. *Journal of magnetism and magnetic materials* **290**, 475-478 (2005).

15 Malinowski, G., Kuiper, K., Lavrijsen, R., Swagten, H. & Koopmans, B. Magnetization dynamics and Gilbert damping in ultrathin Co 48 Fe 32 B 20 films with out-of-plane anisotropy. *Applied Physics Letters* **94**, 102501 (2009).

16 Du, Z., Chen, C., Cheng, F., Liu, Y. & Pan, L. Prediction of deterministic all-optical switching of ferromagnetic thin film by ultrafast optothermal and optomagnetic couplings. *Scientific reports* **7**, 1-11 (2017).

17 El Hadri, M. S. *et al.* Two types of all-optical magnetization switching mechanisms using femtosecond laser pulses. *Physical review B* **94**, 064412 (2016).

18 Xu, Y. *et al.* From single to multiple pulse all-optical switching in GdFeCo thin films. *Physical Review B* **100**, 064424 (2019).
