# Supplementary material for: All optical control of magnetization in quantum confined ultrathin magnetic metals
Source: Sci Rep. 2021 Aug 5;11:15976. doi: 10.1038/s41598-021-95319-6 (PMC8342544; doi:10.1038/s41598-021-95319-6)
Supplement: Supplementary file 2 — Supplementary Information 2. [file 41598_2021_95319_MOESM2_ESM.pdf]

```
% % M3TM model for thickness-dependent "gamma" and "G_0"
% % discrete and finite number of thicknesses are used temporarily.
% % G_0 is phonon and electron coupling
% % gamma=gamma_0*DOS_F ()
% %=====================================================================
close all
clear all
syms t u
% u(1)=Te, u(2)=Tp, u(3)=m
% Ce= gam*Te = gam*u(1);
Cp = 2.33e6;
% phonon heat capacity of Ni in (J/m^3k)
% % {in some references Cp=C(material)-Ce (its is not used in this code
% % since it changes the ODE to a stiff ODE)
% % for Ni: 455-u(1); [from Nat. Mat.]
% % 455 J/KgK is the total heat capacity of Ni}
Tc = 627;
% Curie Temperature of Ni in (K)
R0 = 17.2e12;
% spin-flip ratio for Ni in (s^-1)
gam0= Cp/(5*Tc);
%Nat.mat: Cp=5*gam*Tc in (J/m^3k^2)
Te0=300;
%intial electron temperature (RT in (K))
Tp0=300;
%intial phonon temperature (RT in (K))
m0=1;
%intial normalized magnetization (m=|Mz/Ms|;unitless)
t0=50e-15;
% laser pulse duration in (s)
I=0.028;
% laser pulse fluence: 28mJ/m^2
d=22e-10;


kB = 8.617333262e-5; % eV / K
kBJ = 1.380649e-23; % J / K
hbarEVs = 6.582119569e-16; % eV s
h = 6.62607015e-34; % J s
hbarJs = h / (2 * pi);
JouleToEV = 6.2415093433e18;
eVtoJoule = 1 / JouleToEV;
E0 = 13.6; % eV
E0J = E0 * eVtoJoule;
a0 = 0.53; % Angstrom
a0m = a0 * 1e-10;
%
dosScale = 1/(E0 * a0^3);
dosScaleJ = 1/(E0J * a0m^3);
T0 = E0 / kB; % K
g0factor = (pi * kBJ / hbarJs) * dosScaleJ;
%%-------------------------------------------------------------------------
% Physical parameters assigned to their values:
Vz = 10; % eV
rs = 4 * a0;
Vz = Vz / E0;
rs = rs / a0;
```

```
lambdaW2 = 49.5e-6; % Experimental, Ni, eV^2
lambdaW2J = lambdaW2 * (eVtoJoule^2);
g0factor = g0factor * lambdaW2J;
%.......................................................................
% Use fixed Lz:
Lz = 50;  % AngstrÃ¶m
Lz = Lz / a0;
LzMin = 10;
LzMax = 50;
deltaLz = 0.5;
LzVec = LzMin:deltaLz:LzMax;
LzVec = LzVec/a0;
%.......................................................................
% Use fixed electron tempeature:
temp = 0.005 * T0; % K
% temp=u(1);
temp = temp /(2* T0);
% ......................................................................
% Bulk Fermi wave number and bulk DOS at Fermi energy:
% These are used for scaling purposes in the output
kFbulk = ((9 * pi) / (4 * rs^3))^(1/3);
dosFbulk = kFbulk / (2*pi^2);
% Calculate G0 for a set of film thickness:
fprintf('G_0 will be calculated for a set Lz \n')
for j = 1:length(LzVec)
        if j>1
          for k=0:log10(j-1)
              fprintf('\b'); % delete previous counter display
          end
      end
      fprintf('%d', j);
      pause(.05);
    dos = dosMuFunction(temp,LzVec(j),Vz,rs);
    g0VsLz(j) = g0factor * dos;
    gam(j)=gam0*dos;
    R(j)=R0*dos;

fprintf('\n')
fprintf('G_0 has been calculated for a set of Lz \n')
fprintf('G_0 will be calculated for a set of Te \n')


p0=I/(d*t0);
% incoming laser power=(fluence)/(thickness*pulse width)
% =======================================================================
% % 2TM coupled with magnetization dynamics (M3TM):
% u(1)=Te, u(2)=Tp, u(3)=m
% =======================================================================
eqn= @(t,u)[(-g0VsLz(j)*(u(1)-u(2))+(p0/sqrt(2*pi))*exp(-
0.5*(t/t0)^2))/gam(j)*u(1);...
            -(g0VsLz(j)/Cp)*(u(2)-u(1));....
            R(j)*u(3)*u(2)/Tc*(1-u(3)*coth(u(3)*Tc/u(1)))];

% coupled differentialequation solver:
[t,u] = ode15s(@(t,u)eqn(t,u),linspace(0,25e-12,1000),[Te0; Tp0; m0]);
```

```
% =======================================================================
% plot and save Te, Tp, and m for different thicknesses
% =======================================================================
W = figure('visible','off');
yyaxis left
plot(t/1e-12,u(:,1)/Tc,'b',t/1e-12,u(:,2)/Tc,'g','LineWidth',2);
yyaxis right
plot(t/1e-12,u(:,3),'r','LineWidth',2);
 title('M and T vs. time-Ni- ','FontSize',16)
 yyaxis left
 xlabel('\Deltat (ps)', 'FontSize',16)
 ylabel('T/T_C', 'FontSize',16)
 yyaxis right
 ylabel('|M_z|/M_s', 'FontSize',16)
legend('Te','Tp','m','Location','Northeast')
ax = gca;
ax.YAxis(1).Color = 'b';
ax.YAxis(2).Color = 'r';
ax.YAxis(1).LineWidth = 2;
ax.YAxis(2).LineWidth = 2;
box on
set(gca,'ycolor','b','FontSize',16,'LineWidth',2)
set(gca,'XTick',(0:5:25),'Linewidth',2,'FontSize',16)
% axis tight
 sample_lable= {'Ni'};
Lz_label =
{'10A','10.5A','11A','11.5A','12A','12.5A','13A','13.5A','14A','14.5A','15A','
15.5A','16A','16.5A','17A','17.5A','18A','18.5A','19A','19.5A','20A','20.5A','
21A','21.5A','22A','22.5A','23A','23.5A','24A','24.5A','25A','25.5A','26A','26
.5A','27A','27.5A','28A','28.5A','29A','29.5A','30A','30.5A','31A','31.5A','32
A','32.5A','33A','33.5A','34A','34.5A','35A','35.5A','36A','36.6A','37A','37.5
A','38A','38.5A','39A','39.5A','40A','40.5A','41A','41.5A','42A','42.5A','43A'
,'43.5A','44A','44.5A','45A','45.5A','46A','46.5A','47A','47.5A','48A','48.5A'
,'49A','49.5A','50A'};
        for n=21
        sample_index=1;
        Lz_index=n;
        txt = strcat({' '},sample_lable (sample_index),{' -50 fs-I_0=28mJ/m^2-
L_z= '},Lz_label(Lz_index));
        title_text = strcat('M and T vs. time for',txt);
        title(title_text, 'FontSize',14)
        filename = strcat('M2TM_Lz _variability_', num2str(n));
        end
    saveas(W,filename,'fig')
    saveas(W,filename,'tiff')
    print('-dtiff','-r600',filename)

 close all


end

%%
function dosMu = dosMuFunction(temp,Lz,Vz,rs)
%
deltaK = 0.00001;
```

```
Efbulk = ((9 * pi) / (4 * rs^3))^(2/3);
%
kTop = sqrt(Vz); % Max kz at the top of the confinement well
%
mkzTop = floor(kTop * Lz / pi + 1);
kzVec = zeros(mkzTop,1);
for j = 1: mkzTop
    kzVec(j) = kzFunction(j,Lz,Vz);
end
kmuT = sqrt(muFunction(temp,Lz,Vz,rs) * Efbulk);
kzMlist = kzVec(kzVec<kmuT);
kzM = kzMlist(end);
idx = length(kzMlist);
if kmuT-deltaK > kzM
    dosMu = idx / (2*pi*Lz);
else
    dosMu = (1/(2*pi*Lz))*(idx-1+(kmuT^2-kzM^2)/deltaK^2);
end
end
%%-------------------------------------------------------------------------
function kz = kzFunction(nz,Lz,Vz)
% Vz and Lz are assumed to be dimensionless, scaled with Bohr units
% This function is called in kMu and MuFunction functions.
%
    kTop = sqrt(Vz); % Max kz at the top of the confinement well
%
    kzEqn = @(kz) kz * Lz - nz * pi + 2 * asin(kz /kTop);
    kz_guess = [0 kTop];
    kz = fzero(kzEqn,kz_guess); % allowed kz value for given nz,Vz,Lz
end
%%-------------------------------------------------------------------------
function kMu = kMuFunction(temp,Lz,Vz,rs,maxM)
% Vz, Lz, rs and temp are dimensionless, scaled with Bohr units
% This function is called by muFunction (chemical potential function).
%
    kTop = sqrt(Vz); % Max kz at the top of the confinement well
    nbar = 3 / (4 * pi * rs^3);
%
    kMu_guess = kTop;
    lhs = 2 * pi * nbar * Lz / temp;
    kMuEq = @(x) lhs - auxFunKmu(x,Lz,Vz,temp,maxM);
    kMu = fzero(kMuEq,kMu_guess);
end
function termKmu = auxFunKmu(x,Lz,Vz,temp,maxM)
sum = 0;
    for j = 1:maxM
        sum = sum + log(1 + exp((x^2 - ...
                kzFunction(j,Lz,Vz)^2)/temp));
    end
    termKmu = sum;
end
%%-------------------------------------------------------------------------
function mu = muFunction(temp,Lz,Vz,rs)
% Vz, Lz and temp are assumed to be dimensionless, scaled with Bohr units
%
Efbulk = ((9 * pi) / (4 * rs^3))^(2/3);
%
```

```
maxM = 1;
search = 1;
while search == 1
    kMuGuess = kMuFunction(temp,Lz,Vz,rs,maxM);
    kTest = kzFunction(maxM+1,Lz,Vz);
    if kTest > kMuGuess
        mu = kMuGuess^2 / Efbulk;
        search = 0;
    end
    maxM = maxM + 1;
end
end
%%=========================================================================
```
